# Microbial Biostimulants as Response to Modern Agriculture Needs: Composition, Role and Application of These Innovative Products

**DOI:** 10.3390/plants10081533

**Published:** 2021-07-27

**Authors:** Adele M. Castiglione, Giuseppe Mannino, Valeria Contartese, Cinzia M. Bertea, Andrea Ertani

**Affiliations:** 1 Plant Physiology Unit, Department of Life Sciences and Systems Biology, University of Torino, 10135 Turin, Italy; adelemaria.castiglione@unito.it (A.M.C.); giuseppe.mannino@unito.it (G.M.); 2 Green Has Italia S.P.A, 12043 Canale, Italy; v.contartese@greenhasitalia.com; 3 Department of Agricultural Forest and Food Sciences, University of Torino, 10095 Turin, Italy; andrea.ertani@unito.it

**Keywords:** microbial biostimulant, arbuscular mycorrhizal fungi, plant growth promoting microorganism, plant growth promoting rhizobacteria, synergism, abiotic stress, environment, soil biodiversity, land degradation neutrality, European Biostimulant Industry Council

## Abstract

An increasing need for a more sustainable agriculturally-productive system is required in order to preserve soil fertility and reduce soil biodiversity loss. Microbial biostimulants are innovative technologies able to ensure agricultural yield with high nutritional values, overcoming the negative effects derived from environmental changes. The aim of this review was to provide an overview on the research related to plant growth promoting microorganisms (PGPMs) used alone, in consortium, or in combination with organic matrices such as plant biostimulants (PBs). Moreover, the effectiveness and the role of microbial biostimulants as a biological tool to improve fruit quality and limit soil degradation is discussed. Finally, the increased use of these products requires the achievement of an accurate selection of beneficial microorganisms and consortia, and the ability to prepare for future agriculture challenges. Hence, the implementation of the microorganism positive list provided by EU (2019/1009), is desirable.

## 1. Introduction

Soil fertility, as defined by the Food and Agriculture Organization (FAO), is “the ability of a soil to sustain plant growth by providing essential plant nutrients and favorable chemical, physical, and biological characteristics as a habitat for plant growth” [1]. In recent years, it was observed that global agricultural soil has become seriously degraded. In particular, about 40% of the world’s agricultural soil and 24% of the globe’s productive areas have been subjected to a loss of fertility, production capacity, and biodiversity. These phenomena are mainly due to several different factors, including water and wind erosion, salinity, loss of organic matter, and environmental pollution [2,3].

Considering the continuously growing global population, the diminishing of arable land area, and the depletion of the genetic potential of crops, the implementation of novel agricultural technologies are required. Low environmental impact agronomic solutions, aimed at improving plant resilience to adverse soil conditions, are becoming indispensable in guaranteeing the high demand for food with high nutritional values [4,5].

An interesting turning point for sustainability in the agricultural field was the Farm to Fork Strategy (F2F), published in May 2020 by the European Union, whose purpose is to become climate-neutral by 2050. The COVID-19 pandemic has underlined the importance of a robust and resilient food system able to function under all circumstances, and capable of ensuring access to a sufficient supply of affordable food for citizens [6]. In this context, F2F aims to make food systems fair, healthy, and environmentally-friendly, by accelerating the transition to a more sustainable, productive system in which the dependency on pesticides, antimicrobials, and over-fertilization is drastically reduced. Moreover, F2F supports the increase of agricultural land under organic farming in order to preserve soil fertility and reduce biodiversity loss.

In the last several years, research has strongly focused on the use of agro-ecological principles to minimize potentially harmful chemical inputs and manage ecological relationships and agro-biodiversity [7]. Agro-ecology is based on the conservation of biodiversity, on the strengthening of biological processes, and on the looping of biogeochemical cycles. Fitting with the agroecological principles, is the use of biostimulants, products that are able to not only act directly on plants, but also sustain productivity through the selection and stimulation of beneficial soil microorganisms [8].

Plant biostimulants (PBs) are a new generation of products available on the market, which may be useful for achieving agricultural sustainability policies. They are defined by the EU Regulation 2019/1009 as “products stimulating plant nutrition processes independently of the product’s nutrient content with the sole aim of improving one or more of the following characteristics of the plant or the plant rhizosphere: nutrient use efficiency, tolerance to abiotic stress, quality traits, availability of confined nutrients in soil or rhizosphere” [9,10]. PBs may be composed of substances or mixtures and micro-organisms, therefore they are classified as microbial or non-microbial plant biostimulants [11].

A microbial plant biostimulant consists of a microorganism or a consortium of microorganisms listed in the CMC-7 (Component Material Categories, number 7), which includes four different genera: *Azotobacter* spp., Mycorrhizal fungi, *Rhizobium* spp., and *Azospirillum* spp. 

However, although having regulations and limitations may be useful for guaranteeing food security and quality, the stringency and exclusivity of the positive list may strongly affect the potential benefits of these new products. Consequently, it may be appropriate to consider the reduction of the negative list and the expansion of the positive one with new microbial organisms, assuming scientific evidence can demonstrate and support their safety for both the environment and consumers.

Considering the environmental damage associated with current fertilization practices, a research priority is to optimize plant–microbe nutritional interactions for more sustainable agricultural systems [12]. Several plant growth-promoting rhizobacteria (PGPRs) were demonstrated as able to exert a beneficial effect on plant growth under nutritional and abiotic stress or during the restoration of polluted soils. Moreover, plants could also establish symbiosis with arbuscular mycorrhizal fungi (AMFs), which increases the root surface area for nutrient acquisition [13]. The number of research studies related to the beneficial use of microorganisms is increasing at an exponential rate due to the new technologies, which allow for an accurate selection and understanding of the added value of microbial consortia. The European Biostimulant Industry Council (EBIC), founded in 2011, is attempting to encourage the innovation in this field by requesting operational solutions for their harmonized regulatory treatment on the EU market, including safety requirements and an update of CMC-7 in the new legislation.

The aim of this review was to provide an overview on plant growth promoting micro-organisms used alone, in consortium, or along with organic matrices such as plant biostimulants. Furthermore, we discuss their effectiveness and their role as biological tools to limit the physical, chemical, and biological degradation of soil.

## 2. Plant Growth Promoting Microorganisms (PGPMs) and Their Biostimulant Activity 

Some organisms, such as AMFs or PGPRs, may influence plant physiological mechanisms by exerting a biostimulant action [14,15,16,17,18,19]. The nature of PGPM-plant interaction has been explained through various hypotheses. However, it is still not clear whether this interaction is the evolution of a parasitic or saprobiotic relationship. The only certainty is that the plants are exploited by PGPMs to ensure their own survival [20].

AMFs are fungi belonging to the Glomeromycota phylum, which includes three different classes (Glomeromycetes, Archaeosporomycetes, and Paraglomeromycetes), five orders (*Archaeosporales*, *Diversisporales*, *Gigasporales*, *Glomerales*, and *Paraglomerales*), 14 families, 29 genera, and more than 200 species [21,22]. The ability of the Glomeromycota phylum is related to the colonization of plant roots through endomycorrhizal symbioses. Currently, it is reported that more than 80% of land plants are able to establish beneficial interactions with AMFs [23]. Despite the fact that a large number of AMF species have been identified, only a select few are used in agricultural practices [24]. Accordingly, the available commercial inocula contain species almost exclusively belonging to *Rhizophagus* and *Funneliformis* genera that are generalist symbionts, present in almost all soils and under a wide range of climate zones [24,25]. 

On the other hand, PGPR is a very heterogeneous group of endophytic bacteria, which includes the phyla Proteobacteria, Firmicutes, Actinobacteria, and Bacteroidetes [26]. Among the innumerable genera, *Aeromonas, Arthrobacter, Azospirillum, Azotobacter, Bacillus, Clostridium, Enterobacter, Gluconacetobacter, Klebsiella, Pseudomonas, Rhizobium*, and *Serratia* are the most studied, mainly due to their wide diffusion [27]. PGPRs are found in the rhizosphere and are able to promote plant growth, with recent scientific evidence showing the importance of their role in enhancing soil productivity and tolerance to abiotic stress in plants [28]. 

**Figure 1:**
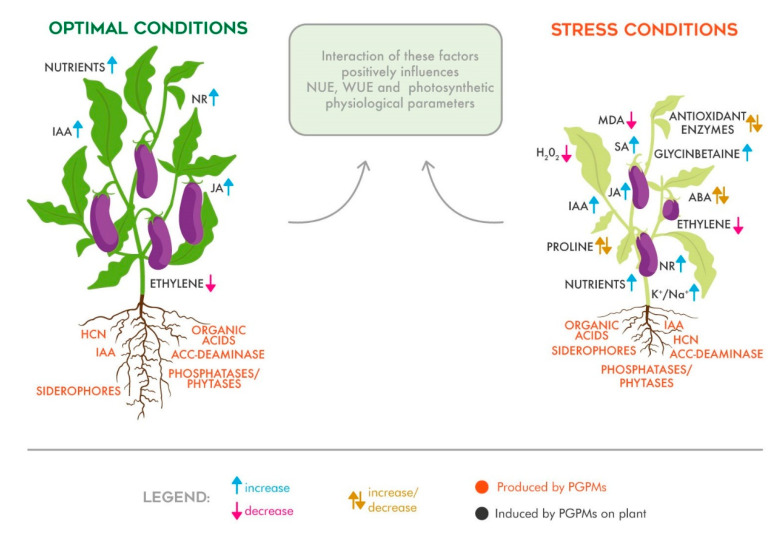
Schematic drawing representing PGPM biostimulant effects under standard and stress conditions. The PGPM inocula and the substances produced by them (in red) induce different responses in plant metabolism (in black), determining the increase (arrow up) or decrease (arrow down) of hormone concentration, enzyme activity, and the K^+^/Na^+^ ratio (up-down arrows indicate discussed data). These metabolic responses positively affect nutrient use efficiency (NUE), water use efficiency (WUE), and photosynthetic physiological parameters.

Although AMFs and PGPRs are finding great success as an innovative agricultural practice, the mode of action by which these PGPMs positively influence crops and soil is still unclear. The reported beneficial effects depend not only on the plant genus or soil typology, but also on the presence or nature of the stress and by the type of inoculum. The hypothesized mode of action by which PGPMs affect plant growth can be classified as follows: (i) hormonal regulation; (ii) balance of cell oxidative status; (iii) water use efficiency and photosynthetic physiological response improvement; and (iv) the improvement of nutrient use efficiency. A schematic representation of the biostimulant effects derived from the inoculation of plants with PGPMs is reported in Figure 1, and will be discussed in the following subsections.

### 2.1. Hormonal Regulation

Among the hormones regulating plant physiological processes, abscisic acid (ABA) plays a key role. ABA acts as anti-transpiration agent leading to a reduction in water loss through the modification of stomatal functioning [29]. Consequently, the importance of ABA is clear, especially in stress conditions caused by a lack of water, increased heat, or salt excess. ABA biosynthesis can be affected by several factors, including the presence of PGPMs in the soil [30,31]. However, the results related to the changes in ABA content after their inoculation are debated. Some studies have reported a higher amount of ABA on plants grown under stress conditions, but not inoculated, as compared to colonized ones [15,29,30,32]. On the contrary, other studies highlighted an increase of ABA in plants grown under stress and inoculated with AMFs [33] or PGPRs [34,35]. In this scenario, the expression of the ABA biosynthetic gene *9-cis-epoxycarotenoid dioxygenase (SlNCED)*, in AMF-colonized tomato plants under stress conditions, suggested that ABA regulation might be affected by the type of stress. Indeed, Duc et al. observed that *SlNCED* was down-regulated in tomato roots colonized by *Septoglomus constrictum* under drought stress conditions, but remained unaffected under standard and heat stress conditions. Moreover, Chitarra et al. demonstrated that, under the same stress conditions, ABA biosynthesis was also affected by the type of mycorrhizal inoculum [15]. 

Indole-3-acetic acid (IAA) is the major endogenous auxin in plants, and is able to regulate several cell processes, including cell elongation and division, root development, and root hair formation [36]. In this context, the relationship of PGPMs with auxins, especially with IAA, is clearer. Although an increase in IAA was observed in plant tissues grown in soils characterized by a lack of water or an excess of salt, a greater increase was recorded in plants that were further colonized by AMFs [33,37,38,39]. Moreover, in the same plants, variations of root morphology and architecture were also observed [37]. This evidence was additionally proven by Liu et al. who studied the auxin pathway in plants grown under drought stress and inoculated with *Funneliformis mosseae.* These authors observed the activation of auxin-related genes (*PtYUC3* and *PtYUC8*), the up-regulation of auxin influx carriers (*PtABCB19* and *PtLAX2*), and the down-regulation of auxin efflux carrier genes (*PtPIN1* and *PtPIN3*) [38].

These scientific evidences suggest that AMFs, by inducing the production of IAA, can amplify the physiological plant response to abiotic stress by causing morphological variations in the root system. From another point of view, since some PGPMs are able to produce IAA, they also represent a potential source of exogenous IAA for plants [16,40,41,42]. 

This peculiar ability to produce and secrete compounds that can be useful to the plant is not exclusively limited to the biosynthesis of IAA. Indeed, some PGPR strains are also capable of producing 1-aminocyclopropane-1-carboxylate (ACC) deaminase, an enzyme that catalyzes the conversion of ACC, the precursor of ethylene, to α-ketobutyrate and ammonia. Consequently, the secretion of ACC-deaminase causes the decrease of ethylene level in both plant and soil, with a concomitant reduction of environmental stress effects on plants [41]. Finally, when PGPRs are able to secrete both IAA and synthesize ACC-deaminase, a cross-talk effect occurs. Indeed, IAA stimulates plant growth, meanwhile ACC-deaminase decreases plant ethylene levels [43].

ACC-deaminase-producing bacteria regulate plant growth by enhancing levels of stress-related hormones, such as salicylic (SA) and jasmonic acid (JA) [34,44,45]. JA is an endogenous regulator hormone that plays an important role in different development processes, since it is involved in key signaling pathways in either biotic or abiotic responses [46]. Some scientific evidence shows that JA biosynthesis is also strictly correlated to AMF symbiosis [47]. Indeed, AMF inoculation significantly increased JA levels in durum wheat, maize, and cucumber plants, both under standard and stress conditions [29,33,47,48]. On the other hand, SA also induces the expression of stress-related genes to maintain membrane stability and prevent oxidative damages [44]. Although the role of SA as a key player in the regulation of the disease signaling pathways is widely accepted [49], this compound also seems to be involved in the symbiotic relationship between PGPMs and plants. When this interaction occurs, SA content significantly increases in the roots, both under drought and salt stress conditions [29,33,45]. In general, the enhancement of JA and SA in inoculated plants alleviates abiotic stress through the synthesis of secondary metabolites [29]. This action mechanism is also confirmed by the decrease of ethylene biosynthesis in roots [47], the increase of IAA [33,42], and the variation in ABA concentration in inoculated plants [30,31].

### 2.2. Balance of Cell Oxidative Status

During stress conditions, both reactive oxygen (ROS) and nitrogen (RNS) species are produced with the aim of regulating a variety of physiological processes to guarantee plant survival [50]. However, since both ROS and RNS are highly reactive molecules, several biomolecules, such as proteins, lipids, and nucleic acids, are susceptible to their strong oxidative activity. Consequently, huge and irreparable damage can be caused to membranes, DNA, RNA, and enzymes, leading to cell death [51]. Different studies reported that plants inoculated with PGPMs had a higher scavenging activity against ROS and RNS [39,40,52,53]. Consequently, a lower amount of hydrogen peroxide (H_2_O_2_) and malondialdehyde (MDA, a marker of lipid peroxidation) are detected in the tissues of inoculated compared to non-inoculated plants [30,32,35,41,48,54]. 

The reduction in ROS levels under different stress conditions could be partly explained by the increase in antioxidant enzyme activity of PGPM-inoculated plants [48]. Indeed, often ascorbate peroxidase (APX), catalase (CAT), glutathione reductase (GR), glutathione peroxidase (GPX), peroxidase (POD), and superoxide dismutase (SOD) activities are substantially enhanced in colonized plants under abiotic stress conditions [29,30,34,41,47,54,55,56]. Nevertheless, as in this case, some exceptions were reported. For example, a down-accumulation and reduced activity of POD, SOD, and GR in mycorrhizal roots of both wheat drought-sensitive cultivar and digit grass were detected [47,48]. Moreover, other studies showed that PGPR treatments significantly decreased CAT, GPX, GR, and SOD activities when inoculated plants were compared to untreated ones [35,57,58,59]. Nawaz et al. suggested that IAA-producing bacteria improved osmolyte accumulation and reduced enzymatic antioxidant activity by accelerating the modulation of the wheat plant biochemical processes [57].

On the other hand, non-enzymatic antioxidants, such as polyphenols, organic acids, vitamins, carotenoids, and glutathione, are also involved in the interaction of PGPMs and plants, influencing their responses to oxidative stress. For example, it was previously highlighted that the accumulation of proline and glycine-betaine may be useful for preventing cellular oxidative damage, in both AMF [29,60] and PGPR [41,57,61] inoculated plants. An exception was reported by Moreno-Galván et al., who described conflicting data regarding proline in PGPR-plant tissues when compared with non-inoculated drought stressed plants [58]. The authors suggested that inoculation could trigger an early proline accumulation in plants, probably by reducing the need for a late accumulation and supporting part of the plant adaptation to drought stress [58]. Moreover, in accordance with Tigka and Ipsilantis, Moreno-Galván et al. also assumed that the plant developmental stage played a key role in the proline accumulation [58,62].

### 2.3. Water Use Efficiency (WUE) and Photosynthetic Physiological Response Improvement

Several studies reported that PGPM application can influence plant water-related parameters by increasing leaf water potential [30,54,63,64], leaf transpiration rate [65], stomatal conductance [30,32,48,54,59,63], and relative water content (RWC) [30,31,35,59], or other factors that may determine positive effects on water use efficiency (WUE) [18,63,65]. For example, Bárzana et al. demonstrated that the roots of AMF-inoculated maize and tomato plants showed a significant enhancement of the relative apoplastic water flow compared to control plants, both under well-watered and drought stress conditions [66]. AMFs have the ability to modulate the switching between apoplastic and cell-to-cell water transport, leading to a higher flexibility in the response of plants to drought or other stress conditions [66]. This phenomenon may be the consequence of a positive modulation of aquaporin genes that consequently enhance the leaf water potential, thus suggesting that AMF plants better regulate their cellular water content [32,67]. On the other hand, the maintenance of a high stomatal conductance allows for a higher CO_2_ uptake during photosynthetic processes. A high concentration of intracellular CO_2_, which is accumulated under stress conditions, negatively affected photosynthesis in *Ricinus communis* [17]. Nevertheless, it was demonstrated that the presence of AMFs significantly reduced intracellular CO_2_ concentration, alleviating the reduction in photosynthetic processes caused by stressful conditions [17]. The effect of PGPM symbiosis on plant photosynthetic activity was further confirmed by the enhancement of photosynthetic rate, not only under drought or salt stress, but also under optimal conditions [15,17,59,65]. Moreover, an increase of the content of photosynthetic pigments was observed in several plant species [17,31,54,56,68]. In particular, chlorophylls seemed to be the most affected [19,41,69,70,71]. It has been hypothesized that PGPM symbiosis neutralizes the negative effect of stressful conditions, counteracting the degradation of photosynthetic pigments, and increasing the maximum quantum yield of open photosystem II [30,35,54,68,71,72].

### 2.4. Improvement of Nutrient Use Efficiency (NUE)

It was demonstrated that the inoculation of PGPMs may contribute to mineral uptake, siderophore production, atmospheric nitrogen fixation, and phosphate solubilization, thus affecting the nutrient use efficiency (NUE) [73]. 

Concerning mineral uptake, it seems that some important elements, such as K, Ca, Mg, Zn, Fe, Mn, and Cu, are better absorbed, whereas the deleterious ones, including Na, are better excreted [29,57]. Different studies have linked the enhancement in mineral uptake to the morphological improvement of root architecture recorded after the inoculation of PGPMs [74,75]. In particular, extraradical hyphae, produced by AMF inocula, could assist plant roots in increasing the soil volume exploited, thus improving the nutrient uptake capacity [19,41,74]. However, these phenomena could also be explained by the over-expression of selective ion channels [76,77], that, in some cases, lead to the improvement of the K^+^/Na^+^ ratio, resulting in an important mechanism, which increases the plant tolerance to salinity [19].

Another possible mechanism by which PGPMs could help plants to effectively absorb nutrients concerns their ability to acidify the soil. Indeed, some PGPMs are able to synthesize organic acids and secrete them in the soil, thus facilitating the solubilization of inorganic phosphates (Pi) and K [78,79,80]. Furthermore, recent studies have shown that they are also capable of producing phytases and acid phosphatases, which enhance the P mineralization [78,79,80]. In addition, hydrogen cyanide (HCN) producing bacteria are involved in increasing P availability and the sequestration of metals with beneficial effects for rhizobacteria and their plant host [81].

Bio-inoculation also increases nitrogen use efficiency through N-fixing microbe activity. The most well-known species are those belonging to the genus *Rhizobium*, which are able to fix the atmospheric nitrogen gas (N_2_) into ammonia (NH_3_). Nevertheless, other species, such as *Azospirillum* and *Azotobacter*, likewise exhibit the ability to fix N_2_ in non-leguminous plants either through a symbiotic or free living relationship [78]. Moreover, Begum, Ahanger, and Zhang found a correlation between increased N-uptake and up-regulation of nitrate reductase (NR) activity in tobacco plants inoculated with AMFs. The higher NR activity allowed for a better N assimilation, thus leading to an improvement of the amino acid and protein biosynthesis [82].

Another important characteristic of PGPMs is the ability to produce ion chelating compounds known as siderophores. Indeed, despite the fact that Fe is one of the most abundant elements present in soil, it is not readily bioavailable since it is available as insoluble Fe^3+^. Consequently, some microbes release siderophores to scavenge iron from the mineral phases with the aim to assimilate it. In this context, plants can benefit from bacterial siderophores to absorb Fe needed for several essential processes, such as photosynthesis [24,83,84,85]. 

## 3. Origin and Selection of PGPMs as Plant Biostimulant Formulation

In the recent years, an increased interest in PGPM-based biostimulants has been observed [86,87,88]. In particular, several studies were conducted employing either native or allochthonous PGPMs, and, in some cases, their biostimulant potential was compared [77,89,90,91,92,93,94,95]. Most of the time, plants are positively influenced by both types of inoculants, which significantly affect their growth by improving nutrient uptake and mitigating oxidative stress [89,92]. In order to find the best inoculation strategies for revegetation and re-establishment of native plant species in degraded ecosystems, autochthonous and allochthonous microorganisms were evaluated. In this context, two recent studies showed a better performance of autochthonous microorganisms, which appear to be physiologically and genetically adapted to the stress conditions of the target environment, compared to allochthonous ones [91,93]. Specifically, since autochthonous microorganisms are able to more quickly and effectively infect the plant root system [90], they appear more suitable to counteract the negative effects derived from stress conditions [77] to improve the uptake and transfer of P, and to stimulate plant growth more so than those deriving from collections [92,93]. However, other studies have demonstrated that native PGPMs are not necessarily better in terms of biostimulant effect when compared to non-native microorganisms. For example, a *Pseudomonas putida* allochthonous strain was particularly efficient as phosphate solubilizing cultivable bacteria [89]. This represented an important feature since the rapid binding of the applied phosphorus into fixed forms is not available to the plants [96]. Moreover, *Bacillus* spp., isolated from Peruvian rainforest soil, significantly promoted the growth of *Arabidopsis*, corn, and tomato plants in a greenhouse [42]. Finally, a commercial *Rhizophagus intraradices*, with the highest colonization intensity in the root system, resulted in an effectiveness comparable to that recorded for native *Glomus* spp. [90]. 

Furthermore, microorganisms isolated from arid and salt-affected soils were evaluated. In particular, evidence showed that the application of PGPMs isolated from degraded areas with salinity and desertification problems could enhance plant tolerance to drought and salt stress [56,59,85]. Likewise, Benabdellah et al. proved that bacterial strains isolated from non-degraded soils showed a stronger response to osmotic stress by producing a higher amount of IAA and proline, resulting in a reduced stomatal conductance in *Trifolium repens* under drought conditions [95]. 

These findings suggest that a better biostimulant effect is not necessarily correlated to the inoculant origins, but mainly to its intrinsic characteristics [19,62] through which the association with the host is more compatible and therefore more beneficial. Santos-Torres et al. supported this hypothesis, pointing out how the inoculation of the same strains (*Rhizobium* sp. T88 and *Herbaspirillum* sp. AP21) on two different host plants (ryegrass and red clover) produced different effects. In particular, they observed the highest plant growth in ryegrass inoculated by *Rhizobium* sp. T88 and the greatest growth promotion in red clover infected by *Herbaspirillum* sp. AP21 under a P-deficiency soil stress condition. Moreover, two different mechanisms to improve P-availability were proposed and demonstrated on the basis of the inoculum and plant association [79]. A similar experimental design was employed by Nawaz et al. [57]. In particular, they evaluated the effects derived from the inoculation of three different halotolerant PGPR strains (*B. pumilus*, *Exiguobacterium aurantiacum*, and *P. fluorescens*) on salt tolerant and salt sensitive wheat varieties. The authors discovered that *B. pumilus* and *E. aurantiacum* displayed a more evident impact on the salt resistant genotype, whereas the growth and yield improvement of the salt sensitive genotype was more influenced by *P. fluorescens* [57].

For these reasons, it was established that the selection of adequate strains to be inoculated in the soil is an issue of utmost importance to increase the success of bio-inoculation under environmental stressful conditions [97].

The achievement of higher production and quality may have different needs in terms of inoculum type when compared with the aim of making saline and drought soils productive, or compared to the reforestation and re-establishment of native plants in degraded soil. Thus, according to the conception of the microbial biostimulant application, it could be more effective to select some genera than others.

Based on these considerations, it should be essential to select those PGPMs that are able to form the largest number of symbiotic relationships, under the most varied environmental conditions. However, due to the elevated genetic variability of PGPMs, further investigation aimed at profiling the characteristics of each inoculum, and the possibility of utilizing different PGPM genera in plant biostimulants, are needed in order to gain the most appropriate multispecies consortia or single strain based on the aim pursued.

## 4. Synergistic/Additive Effects Between Microbial Biostimulant Product Components 

Green deal purposes, combined with the guidelines of the European Regulation 2019/1009, aimed to reduce the use of chemical fertilization via different strategies. Among them, the improvement of biostimulant efficiency was taken into consideration. In order to find the appropriate tools capable of exerting the most effective biostimulant activity on plants, many studies focused on the triple interaction of plants, PGPRs, and AMFs [98,99]. The potential beneficial effect of the combination of different types of inocula as plant biofertilizers may depend on their better stability in adverse environmental conditions [100]. Recent studies have shown a better biostimulant activity when different substances were combined together [101,102,103], demonstrating synergistic or additive interactions [103]. In this context, microbial-based biostimulants can be formulated using: (i) a single PGPM strain; (ii) an AMF/PGPR multispecies consortia; and (iii) a combination of PGPMs with organic and inorganic chemicals. In the following section, the synergistic or additive effects derived from the use of different microbial inocula, along with other substances, will be described.

### 4.1. Biostimulant Effects Resulting from Co-Inoculation of AMFs and PGPRs

PGPRs may act not only as biostimulant agents, but, in the presence of AMFs, they can also behave like a mycorrhizal helper (MH) [24], especially if strictly associated with their mycelium and spores [104]. Vice versa, AMFs can also enhance the activities of nitrogen fixing and phosphorus solubilizing bacteria [105]. For example, MH activity may be explained by the fact that, under simultaneous inoculations with AMFs and PGPRs, the AMF colonization sharply improved in Arizona cypress seedlings [106], in litchi tree [107], and in the common bean [108]. Root colonization might be boosted by PGPR capability to produce cell wall-degrading enzymes that facilitate AMF establishment. Moreover, PGPRs can also release a large variety of secondary metabolites, which are able to enhance the root exudation rates, resulting in higher cell permeability and hyphal growth increase [107,109]. The employment of a mixture of AMFs and PGPRs is not only a potential plant growth promoter tool, but also a beneficial condition for both microorganism groups. 

Consequently, several studies investigated the combined use of PGPRs and AMFs as biostimulant agents (Table 1). The biostimulant effectiveness of a great variety of consortia, containing both plant aiding bacteria and AMFs, was tested with different plants and growing conditions.

An enhancement of plant biomass and yield was detected in Arizona cypress, chickpea, wheat, swamp oak, Jerusalem artichoke, and pea plants when inoculated with PGPRs and AMFs, often exhibiting better results than non-inoculated plants or plants inoculated with a single strain [98,99,106,110,111,112].

Furthermore, a recent study conducted on wheat plants by Dal Cortivo et al., highlighted a significant increase of specific gluten protein subunits involved in dough strength and elasticity [110]. The upregulation of protein subunits was triggered particularly by a biofertilizer containing *R. irregularis* together with *B. megaterium* and *Frateuria aurantia*. Moreover, in the study conducted by Laranjeira, the highest crude protein content in chickpeas, inoculated with AMFs together with PGPRs, was observed [98].

Other investigations highlighted how synergic inocula can improve the quality of crops, as Nacoon et al. reported [112]. Their study described a greater increase of inulin content in Jerusalem artichoke inoculated with *Klebsiella variicola* and *R. intraradices*. In particular, the authors reported that inulin content was significantly higher in comparison to the non-inoculated control, fertilized control, and single strain inoculated plants. Considering that the individual factors showed only partial effects on inulin production, the authors underlined a positive plant response to the co-inoculum [112].

On the other hand, this synergism can also protect plants from different abiotic stresses, as demonstrated by the modulation of proline, H_2_O_2_, MDA, and ROS scavenger compounds [106,111,113,114]. For example, Barnawal observed a reduction of ACC content in dual-inoculated plants dealing with a reduction of ethylene-related stress response. In this study, the ACC-oxidase activity was induced by *Arthrobacter protophormiae*, whereas the activity of the ACC-synthase was reduced by both *R. leguminosarum* and *Glomus mosseae*, resulting in a 60% ACC reduction [111]. Moreover, the same authors highlighted that ACC-deaminase was strongly affected by inocula [111].

The potential capacity of dual-inocula to help plants in overcoming stress conditions is not exclusively linked to ethylene-related pathways, as suggested by Moreira et al. Indeed, these authors, by evaluating the effect of the dual-inoculation on maize grown under salt stress conditions, reported an increase in K uptake and better Na exclusion activity [115]. In agreement with this study, an effective improvement of the nutrient uptake was also observed by Dal Cortivo et al., who evaluated the potential effects derived from the inoculation of two different AMF and PGPR consortia. In particular, the authors described that both consortia were able to enhance the uptake of low-mobile nutrients (Ca and Zn). However, they also highlighted that the consortium composed of the association of *R. irregularis* and P and K solubilizing bacteria (*B. megaterium,* and *F. aurantia*) displayed the best plant growth and nutrient uptake responses. This could be explained not only by a better P and K availability, but also by a stimulating effect on the cyanobacteria activity, which were shown to alleviate nitrogen deficiency in wheat roots [110].

The capabilities of the dual-inoculum to improve the nutrient uptake would allow for the reduction of chemical fertilizers [116], the replacement with cheaper sources of nutrients [112], or the avoidance of their use.

### 4.2. Biostimulant Effects Derived from the Inoculation of PGPMs along with Biologically Active Matrices

In order to maximize the efficiency of microbial-based biostimulants, different formulations containing PGPMs and biologically active matrices, including plant exudates, protein hydrolysates, humic acids, agro-food or industrial by-products, composts or compost extracts, sewage, algae or algae extracts, were evaluated [117].

#### 4.2.1. PGPMs in Combination with Compost

Compost and vermicompost are considered an important raw material for biostimulant formulations. Although, according to the EBIC definition, they are not considered biostimulants, the bioactive compounds that can be potentially extracted from them (i.e. humic substances, phytohormones, and amino acids, etc.) show interesting properties [118]. Several investigations reported that compost and PGPMs, when mixed, are able to determine a positive synergistic effect on plant growth. For example, a significant and positive effect was observed on basil growth after the inoculation of a halotolerant bacterial strain (*Dietzia natronolimnaea*) and a mycorrhizal fungus (*G. intraradices*) amended with vermicompost [119]. This synergistic effect was also confirmed by Ait-El-Mokhtar et al. when, after AMF inoculation along with green waste compost, they observed an improvement of salt tolerance on date palm, as proven by the increased K^+^/Na^+^ and Ca^2^^+^/Na^+^ ratios [120]. Moreover, the authors suggested that the beneficial effects on plant growth could be linked to a more efficient acquirement of nutrients released by the compost due to the presence of AMF. Consequently, the highest improvement of K, P, N, and Ca intake was recorded when AMFs were inoculated in presence of compost, whereas it was less evident if the plants were separately treated with AMFs or compost [120]. The authors also highlighted the enhancement of N uptake due to the dual treatment and an increased quantity of Mg in plant tissues, probably resulting in an improved plant photosynthetic capacity [120]. Finally, a smaller increase in MDA and H_2_O_2_ levels under salt stress, improvement of antioxidant enzyme activities and proline content, indicated that the application of compost and AMF was the most efficient way to boost the tolerance to salinity [120], thus suggesting a synergistic effect in modulating plant physiological and biochemical processes. 

#### 4.2.2. PGPMs in Combination with Humic Acids

Humic acids are the brown-black, polymeric, alkali-soluble acids found in soils, plants, seagrasses, fungi, sediments, terrestrial, and marine waters [121]. They contribute to soil fertility and influence the agricultural environment by complexing with metals and organics, which can modify the toxicity of heavy metals, pesticides, and herbicides [121]. It was demonstrated that the combined application of humic acids with PGPMs was able to affect plant performances. For example, a biostimulant formulation containing three diazotrophic endophytic bacteria strains and humic acid-like substances, showed a positive influence on sugarcane yield [122]. To explain the observed effects, Da Silva hypothesized that the antioxidant enzymes activated by humic acids, and osmoprotectant PGPR-induced mechanism, were combined when the components are used together, showing a boosted biostimulant effect [122]. By means of a similar mechanism, Torun et al. also explained that the application of AMFs and K-humate mixture on olive trees, led to a reduction of H_2_O_2_ content, protecting the lipid membrane from oxidative peroxidation [123]. In addition, AMF and humic acid application promoted the maintenance of leaf water status, chlorophyll fluorescence, and total phenolic content [123]. By evaluating the integrated effects of two *Bacillus* strains (N_2_-fixing and P-solubilizing) and humic acids, separately or in combinations, Ekin et al. showed that a combined application of PGPRs with humic acids, produced the best response in terms of growth, tuber yield, and nutrient content of potatoes under field conditions [124].

#### 4.2.3. PGPMs in Combination with Algae Extracts

Algae extracts are considered an important non-microbial raw material for plant biostimulant formulation. In particular, they are able to induce positive effects on plant performance because of their bioactive compound content [125]. A number of studies showed that algae extracts are a rich source of carbohydrates and proteins, lipids, key amino acids (such as arginine and tryptophan), vitamins, osmolytes (such as proline and glycine betaine), and several plant hormones (such as indolebutyric acid, phenylacetic acid, auxin, gibberellins, cytokinins, polyamines, trans-zeatine, and kinetin, etc.) [126]. Furthermore, it was reported that seaweed extract-based biostimulants had an interesting beneficial effect on the activation of native soil microorganisms, including saprophyte bacteria, fungi, and PGPRs [8].

The application of an AMF microbial-based biostimulant containing *R. intraradices* and seaweed extracts on tomato plants positively stimulated plant growth and yield in a different, but complementary manner [127]. In this experiment, when AMF was inoculated alone, the treatment enhanced leaf development and early flowering, but also caused a decrease in protein biosynthesis, carbohydrates, and lipids. On the contrary, the application of seaweed extracts alone enhanced root development and protein content. Their combined application showed an additive effect (in leaf and root growth, and protein and carbohydrate content), but also a synergistic effect on tomato plants, resulting in an earlier flowering and AMF colonization when compared to single treatments [127]. Similar effects were observed when PGPRs (*Bacillus licheniformis*, *Bacillus megatherium*, *Azotobacter* sp., *Azospirillum* sp., and *Herbaspirillum* sp.) were inoculated with water algae (*Chlorella vulgaris*). In this study, Kopta et al. showed that bacterial-algal preparation significantly affected fresh weight, carotenoids, and total antioxidant capacity of lettuce plants under heat stress conditions in comparison to untreated control plants [128].

#### 4.2.4. PGPMs in Combination with Silicon (Si)

The enhancement of the biostimulant effect on plants caused by a potential synergic relationship was also detected with AMFs and inorganic micronutrients. For example, in strawberry plants an increase of biomass was recorded after the inoculation of *R. clarus* coupled with Silicon (Si), under both standard and drought-stress conditions [129]. In this context, Si promoted AMF colonization and the formation of fungal structures, while AMF increased the uptake of Si from plants. Moreover, an improved uptake of Zn and Fe, an increased expression of the genes coding for enzymes involved in the antioxidative defense system, and an elevated water uptake capacity and WUE, likely related to the synergy between AMF and Si, were observed [129]. In particular, the intake of Zn and Fe seemed to be affected by Si application and is able to induce to the up-regulation of genes encoding for the Fe transporters (IRT1 and IRT2) belonging to the ZIP (Zrt/IRT-like protein) family, which also includes Zn transporters [130]. On the other hand, the cellular oxidative status can also be strongly affected. This beneficial effect could be either due to the ability of Si to increase the production of secondary antioxidant metabolites [131] or due to the regulation of the enzymatic activity or gene expression of antioxidant enzymes, such as superoxide dismutase (SOD), catalase (CAT), and glutathione peroxidase (GPX) [132].

#### 4.2.5. PGPMs in Combination with Amino Acids

Biostimulant formulations based on amino acids are taken under consideration for their beneficial action on plant growth and production quality, especially when environmental stresses occur [133,134]. Among the different properties of amino acids, these compounds are considered to be metal ion chelators, anti-stress factors, and influencers of photosynthetic system and hormone metabolism [135]. Amino acids can be obtained from both plant or animal proteins by chemical or enzymatic hydrolysis. 

In this context, a recent study evaluated the application of L-tryptophan, the aminoacidic precursor of auxins, along with different PGPRs isolated from irrigated fields, semi-arid, and arid regions [31]. The application of bacterial strains (*B. cereus*, *B. pumilus*, and *Pseudomonas* sp.), isolated from a moisture-deficit area, were able to provide higher IAA, gibberellic acid (GA), and ABA content and a lower decrease in RWC. This phenomenon, enhanced by the additional application of L-tryptophan, allowed for the maintenance of a good phytohormone ratio under drought stress conditions, limiting the deleterious effects of abiotic stress. Furthermore, better water conservation was also observed in the treatments in which PGPRs selected from moisture-stressed areas were inoculated in association with tryptophan. As a consequence, root and shoot dry weight of the inoculated plants were higher, thus suggesting a better development of these organs [31].

#### 4.2.6. PGPMs in Combination with Cell-Free Culture Supernatant (CFCS) and Exopolysaccharides (EPS)

An additional strategy to improve plant growth and abiotic stress tolerance could be the combination of the PGPMs with their liquid culture in order to enhance PGPM field performance and extend their shelf-life in the soil [136]. For example, the combined application of *Bradyrhizobium* living cells with their cell-free culture supernatant (CFCS) and metabolite exopolysaccharides (EPS) were evaluated on pigeon pea [136]. *Bradyrhizobium* is known for its nitrogen fixation ability, phytohormone and siderophore production, phosphate solubilization capacity and exopolysaccharide synthesis [137]. Although, these features make this bacterium a good performer in terms of plant growth promotion, the best results were obtained with the mixture of the three components. The sole application of CFCS played a significant role in growth promotion through benzimidazole antioxidant activity and nodulation. Moreover, the increase of ascorbic acid, pantoic acid, and benzoic acid was recorded, suggesting that the application of CFCS made stronger the symbiotic association between plants and PGPR. The authors hypothesized that the positive effects exerted by EPS could be due to its functions as a protective coat for the inoculated PGPR and as carbon source useful for the improvement of root colonization, biofilm, and nodule formation. Based on the characteristics of each component, the best performance, in terms of plant growth, was observed when *Bradyrhizobium* was inoculated with CFCS and EPS. In particular, this formulation better contributed to supporting the growth of indigenous soil rhizobia then the sole inoculum [136]. 

## 5. Antagonistic Effects Resulting from PGPM Co-Inoculation 

Despite scientific evidence highlighting that synergistic or additive effects of PGPM co-inoculation can effectively occur, there are some exceptions. Indeed, co-inoculants do not have universal positive plant-growth promoting functions, but their application can also produce antagonistic interactions among the inoculated PGPMs, thus leading to a reduction of the expected effect [138,139]. 

For example, root-associated *Bacillus* and *Pseudomonas* species may show antagonistic activity against other beneficial bacteria and fungi [139,140]. Moreover, Couillerot et al. reported an inhibitory action *of P. fluorescens* towards *Azospirillum brasilense*, which was ten-fold less abundant on roots. The co-inoculation of the mixture on wheat plants showed a plant growth promotion capacity similar to single inoculations, but the authors concluded that this effect could be exclusively linked to *P. fluorescens* inoculation [141]. 

An additional antagonistic effect was observed between two beneficial *Pseudomonas* species when co-inoculated [142]. In this case, *P. simiae* established significantly higher population densities than *P. fluorescens* by inhibiting the growth of *P. fluorescens*. Moreover, despite the fact that no significant effect on root fresh weight was observed, the combined inoculation of *P. simiae* and *P. fluorescens* on *A. thaliana* roots resulted in a lower shoot fresh weight in comparison to the inoculation of the single microorganism [142].

Antagonistic interactions were also observed when fungal strains were co-inoculated [139]. For example, the co-inoculation of *R. irregularis* and *G. aggregatum* resulted in the abundance reduction of both fungal species [143].

Therefore, PGPM consortia do not necessarily produce an additive or synergic effect, but in some cases a decrease in the biostimulant effect can be observed after co-inoculation [144]. 

In conclusion, the efficacy of a microbial mixture cannot be predicted by the simple evaluation of the performance of a single strain but, in order to obtain a successful biostimulant formulation, it would be appropriate to consider the compatibility among the different strains employed [139]. 

## 6. Influence of PGPM Inoculum in Improving Nutritional and Nutraceutical Aspects on Fruits

In recent years, consumers showed an increased interest in plant food grown in an eco-sustainable way that is free from synthetic chemicals, fertilizers, and pesticides [145]. This new trend in finding and eating environmental-friendly fruits is mostly linked to the negative perception concerning the use of phytochemicals and chemical fertilizers in traditional agricultural practices. Indeed, despite chemical fertilizers allowing for the obtainment of greater agricultural production, the fruits harvested from plants treated with these products were shown to not only to have worse quality, but also negative effects on human health [146]. Consequently, many people have started to consider organic foods over conventional ones because they are perceived as healthier and less prone to chemical exposure. In particular, consumers believe that eating organic food helps to reduce stress levels, maintain an energetic lifestyle, and avoid the side effects derived from the indirect chronic intake of phytochemicals [147].

In this context, the treatment of plants with biostimulant formulations may positively affect plant growth by regulating the uptake of nutrients and water or activating specific enzymatic and non-enzymatic systems [9,28,148]. Furthermore, in several cases it was shown that biofortification is effective not only in obtaining a faster and larger agricultural yield, but also in increasing the product quality [145]. However, this aspect has been investigated only recently, and a number of results reported in the scientific literature suggest that the potential effect derived from the application of biostimulants may depend not only on the type of formulation, but also on the plant genus [5,149].

Here, we used a meta-analytical approach aimed to analyze the most influential qualitative parameters affecting the commercial value of plant foods. Size and weight are the first parameters that are examined by consumers that preferentially choose larger fruits [5,149], while, from a nutritional point of view, the content of sugars and proteins are the most important. Indeed, the sugar content affects the taste of fruit, thus modifying consumer satisfaction. Meanwhile, plant foods with high protein content are very appreciated, due to the limited distribution of these macromolecules within the plant kingdom [150]. Finally, the modern consumer is also interested in foods with enhanced nutraceutical properties. In particular, in the recent years an increase in the purchase of foods with a higher content of bioactive compounds, which can have beneficial effects on human health, including antioxidant properties, was reported [151].

**Figure 2:**
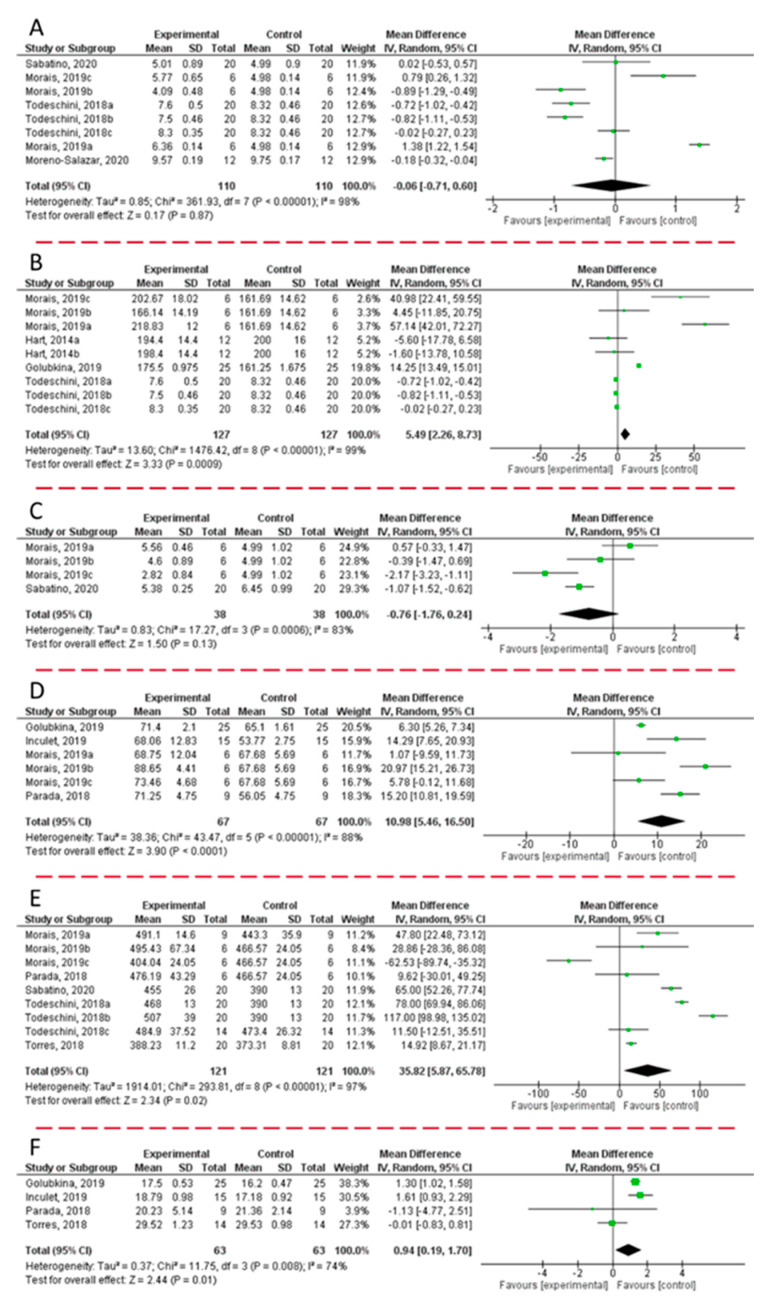
Forest plot representation of the effects derived from biostimulant application on weight (**A**), content of sugars (**B**), proteins (**C**), polyphenols (**D**), anthocyanins (**E**), and antioxidant property (**F**) of fruits harvested from plants treated with microbial inoculation. Data were extrapolated from [152,153,154,155,156,157,158,159,160], and plotted according to the mean difference. The weight of each study is represented by the size of the green box. The forest plot displays a horizontal line that indicates the lower and upper limits of the 95% confidence interval (CI) of the effect reported for each study. The vertical line represents the no-effect, and if it crosses the horizontal lines it indicates the non-significance of the data reported in the respective study. The black diamond at the bottom represents the average effect size. Heterogeneity was assessed using I_2_ statistical test that represents the amount of total variation that could be attributed to heterogeneity.

Data were collected from scientific papers that exclusively satisfied all the pre-fixed inclusion criteria. Briefly, the previously published articles (*n* = 180) were obtained by a literature search on PubMed, Scopus, Google Scholar, and ISI Web of Science research tool, using the following keywords: “biostimulant(s)” OR “microbial inoculation(s)” OR “inoculation(s)” AND “food quality” OR “quality”. Then, a manual screening of the articles was performed by simply reading the title, abstract, or full text. Original articles were included exclusively if they met the following inclusion criteria: (i) the language should be English; (ii) articles should be published in peer-reviewed journals, (iii) after being reviewing by experts, (iv) within the last 7 years; (v) the study design should be randomized controlled clinical trials, (vi) in comparison to untreated control group; (vii) the intervention should be the application of a formulation containing microbial inoculation; (viii) only studies in which the number of replicates was clearly reported should be included; (ix) the measurement outcome should be weight/yield, sugar content, protein content, polyphenol content, anthocyanin content, and antioxidant activity measured by DPPH assay; and (x) when outcomes at different time points were presented in the study, the longest follow-up duration was exclusively selected. Consequently, from the 180 published full text articles identified during the literature search, 164 were excluded. Data from the selected articles (*n* = 9) were employed for the meta-analysis. Figure 2 displays the forest plot analysis, which reported the changes observed in weight (Figure 2A), the content of sugars (Figure 2B), proteins (Figure 2C), polyphenols (Figure 2D), and anthocyanins (Figure 2E), and the antioxidant activity (Figure 2F) of fruits harvested from plants treated with microbial biostimulants.

Since data were obtained from studies, which were independently performed, the forest plots displayed in Figure 2 were built using random effect, in accordance with the high heterogeneity values calculated between the selected studies. Statistical heterogeneity among studies was checked by the Cochran Q test (with a significance level of *p* < 0.05) and the I^2^ statistics. Furthermore, sensitivity analyses were performed to evaluate the influence of each study on the overall effect size. Finally, potential publication bias was excluded by visual inspection of the respective funnel plot. 

Although the analysis of the six forest plots evidenced heterogeneity (I^2^ > 75%), publication bias was not detected. Since the various studies take into consideration the application of formulations based on different microbial inoculations on various plant genera, the high heterogeneity observed is more than understandable. As a general trend, the combined results of the selected articles from the random-effect model suggested a significant effect of the microbial inoculation on the observed parameters, except for fruit weight (WMD: −0.06; 95% CI: −0.71, +0.60; I^2^ = 98%; *P* = 0.87) and protein content (WMD: −0.76; 95% CI: −1.76; +0.26; I^2^ = 83%; *P* = 0.13). On the other hand, the most positive results were related to the content of sugars (WMD: +5.49; 95% CI: +2.26, +8.73; I^2^ = 99%; *P* = 0.0009), polyphenols (WMD: +10.98; 95% CI: 5.46, 16.50; I^2^ = 88%; *P* = 0.0001), and anthocyanins (WMD: +35.82; 95% CI: +5.87, +8.73; I^2^ = 97%; *P* = 0.02). Consequently, an increase of the antioxidant activity was also recorded (WMD: +0.94; 95% CI: +0.19, +1.70; I^2^ = 74%; *P* = 0.01).

## 7. Microbial Biostimulants as a Solution to Limit Land Degradation and Unsustainable Agriculture 

Land degradation is defined as anthropogenic processes resulting in the decline or loss of biodiversity, ecosystem functions, and ecosystem services [161]. Soil degradation is usually recognized in six biophysical processes: water erosion, wind erosion, excess of salt, chemical degradation, physical degradation, and biological degradation [162]. All these processes are mainly caused by unsustainable agricultural practices, including incorrect land or water management [163] and the abuse of chemical fertilizers [164]. These processes have an immediate on-site impact, rendering the lands pauperized in quality and unable to support plant growth [162].

In the last century, biological degradation was rarely considered, while other factors, such as the lack of water and nutrients, were the main focus of scientific research. However, recently it was observed that biological degradation, including the depletion of the microbial component and the reduction of microbial diversity, also strongly influences agricultural yields [165,166]. 

Among the main causes of biological soil degradation, soil organic carbon pool impoverishment [167], soil pH [2,168,169], monocropping [2], and adverse climate conditions are the most important [170,171]. Indeed, the decrease of soil pH determines the minor microbial nutrient availability and the reduction of soil biological activity, leading to a decrease in the more sensitive and rare species, and limiting plant growth [2,168,169]. On the other hand, continuous monocropping causes soil depletion, leading to a decrease of beneficial microorganisms, an impoverishment of the soil microbial community structure, and an increase in pathogen presence [2]. Finally, adverse climate change, including enhanced rainfall, significantly reduces the species richness of soil bacteria and fungi [170,171]. Furthermore, erosion and variable warming reduce the network complexity of soil microbiomes [171,172]. This implies the reduction of decomposition activity and nutrient cycling, as well as resource availability. These factors limit the microbial resilience to environmental stresses by causing long-term adverse effects on soil functions [172].

Microbial functional diversity largely influences important soil processes (e.g., production of NO_3_, and fluxes of N_2_O and CH_4_), and the loss of soil microbial diversity results in a decline of specialized soil functions followed by a decline in the important consequences of terrestrial ecosystems [173]. The importance of a highly diversified microbial component was confirmed by its key role in the C cycle and in the development of soil organic matter (SOM). In fact, the microbial community and microbial byproducts are a strong driver of SOM production and heterogeneity [174,175], contributing to form more than half of the organic carbon in soil through the production of microbial necromass [176,177].

In addition to the role of soil microbiota in the cycling of elements, and in the stabilization of soil structure, elevated soil microbial activity is indispensable for efficient crop production, the ability to maintain healthy plants, and ensuring a good yield under different environmental conditions. Global environmental changes can compromise both plant and soil biodiversity, suggesting a complex feedback between plants and microorganisms under stressed environmental conditions [165,178]. Several studies demonstrated that climate change decreased plant diversity and yield, and a more negative effect was observed under reduced soil biodiversity [165]. This happens independently from plant genotypes, indicating that the negative effect of soil biodiversity loss could generally come from soil microbes [165,179]. In support of this evidence, plant growth under high microbial diversity displayed higher productivity and greater recovery under stress conditions. Moreover, the yield losses were mitigated in the presence of elevated soil microbial communities, suggesting their potential and crucial role as yield stabilizers after global change disturbances [179].

Furthermore, evidence highlights the importance of the microbial component and their diversity not only for crop management, but also as a promising biological tool to recover degraded soils and implement revegetation activities [91,92]. For example, a bacterial consortium (*Azospirillum* spp., *Azoarcus* spp., and *Azorhizobium* spp.) and two AMF-PGPR consortia (*Rhizophagus irregularis* and *Azotobacter vinelandii*, and *R. irregularis*, *Bacillus megaterium*, and *Frateuria aurantia*), inoculated wheat demonstrated a general increase in total microbial biomass and soil enzymatic activities. These findings suggest an enhanced microbial metabolism, mainly observed when the inoculum contained both PGPRs and AMFs [110]. In particular, the consortia composed of *R. irregularis*, *B. megaterium*, and *F. aurantia*, stimulated the cyanobacteria growth, which were then more able to produce a higher amount of plant growth-promoting substances. Similarly, the bacterial consortium stimulated the abundance of bacteria belonging to the Flavobacteriaceae family, which plays an important ecological function in terms of organic matter turnover [110]. Baldi et al. also reported on how AMF influenced soil biodiversity by enhancing soil microbial biomass up to 53% in an apricot orchard [180].

A concrete answer was given by the land degradation neutrality (LDN), proposed by the United Nations Convention to Combat Desertification (UNCCD), which set out the ambition to maintain or increase the amount and quality of land resources by compensating for any land degradation with land restoration [163]. The only way to achieve LDN and sustainable land management (SLM) is by ensuring available instruments are able to maintain proper agricultural input use. Among these strategies, biostimulant products can be considered a powerful tool. Based on previously reported data, microbial biostimulants could be an efficient tool for satisfying the goals of LDN, SLM, agricultural productivity, and resettlement processes of soil biodiversity. 

## 8. Conclusions 

Research on sustainable agronomic tools, which are able to improve plant resilience to adverse soil conditions and ensure agricultural yield, is in progress. Recently, it was observed that biological degradation, including the depletion of microbial components and the reduction of microbial diversity, negatively affects yields. Based on the considered studies, microbial biostimulants seem to be both an adequate response to the poor bio-functions of degraded soils and able to satisfy the goals of agricultural productivity, LDN, SLM, and re-establish soil biodiversity.

The inoculation of a single PGPM strain appears to be effective as a plant biostimulant. Nevertheless, microbial biostimulants based on AMF and PGPR multispecies consortia and formulations based on PGPMs in addition to organic matrices, seem to be a better option when compared to the single strain application. Indeed, these mixtures can exert synergistic or additive biostimulant effects. 

The selection of microbial biostimulant product components play a key role on the formulation efficacy. The intrinsic characteristics of the selected inoculants, their associations with host plants, and the interaction between PGPM strains along with organic matrices, can make the difference with regard to the biostimulant activity of each formulation. The large variability in biostimulant effects and effectiveness allows them to satisfy more diverse needs, such as soil fertility, degraded soil recovery, and preserving yield. Due to the elevated genetic variability of PGPMs, different approaches can be considered. First of all, it could be useful to select PGPMs, which are able to establish the largest number of relationships with plants and rhizospheres to be used under the most diverse of environmental conditions. Moreover, further investigations are focused on selected microorganisms with specific activities to satisfy particular aims, such as the improvement of uptake in the case of the low availability of nutrients; the use of species-specific PGPMs to re-establish native plants in degraded soils; and the application of PGPMs isolated from areas with salinity and desertification problems to enhance plant tolerance to drought and salt stress, etc. On the basis of the above-mentioned approaches, the research on microorganism application could help to prepare for future agriculture challenges.

Nevertheless, investigation on microorganisms was not encouraged because of their lack of consideration in applicable legislation. For the first time, the new European Regulation (EU) 2019/1009 included microorganisms within fertilizer legislation, though, to date, the authorized positive list is rather restrictive. Considering the beneficial effects of the application of PGPMs on parameter-related fruit quality, such as weight, content of sugars, proteins, polyphenols, and anthocyanins, and antioxidant properties, an amendment of the list is desirable in order to provide farmers with more techniques to safely feed the increasing population, while concurrently respecting the environment.

## Figures and Tables

**Table 1:** AMF and PGPR consortia and their additive/synergistic biostimulant effects on inoculated plants.

AMFs/PGPRs Consortium	Host Plant	Abiotic Stress	Additive/Synergistic Effect	REF
*Rhizophagus irregularis*, *Funneliformis mosseae*, *Pseudomonas fluorescens*	*Cupressus arizonica* Green	Drought	Enhancement of plant growth parameter; increase of APX and GPX enzymes activities; decrease of H_2_O_2_ and lipid peroxidation; and alleviation of water-deficit damage and improvement of drought tolerance	[106]
*Arthrobacter protophormia*, *Rhizobium leguminosarum*, *Glomus mosseae*	*Pisum sativum* L.	Salt	Improvement of plant weight; decrease of proline content and lipid peroxidation; increase of pigment content; enhancement of nutrient uptake; alleviation of salt stress; and enhancement of AMF colonization and nodulation	[111]
*Claroideoglomusetunicatum*, *Acaulospora* sp., *Rhizobium* sp., *Burkholderia* sp.	*Schizolobium parahyba* var. *amazonicum*	-	Improvement of absorption of chemical fertilizers; and enhancement of wood yield	[116]
*Rhizophagus irregularis*, *Bacillus megaterium*, *Frateuria aurantia*	*Triticum aestivum* L.	-	Improvement of low-mobile nutrient uptake (Ca and Zn) and enhancement of nutrient uptake responses; increase of plant growth; enhancement of total microbial biomass and microbial metabolism; and increase in gluten quality	[110]
*Pantoea agglomerans*, *Bacillus* sp., *Rhizophagus fasciculatus*, *Rhizophagus aggregatum*	*Casuarina obesa* Miq.	Salt	Increase of survival rate of plants compared to control plants; improvement of frequency of mycorrhization; enhancement of chlorophyll and proline content; and higher resistance to salinity	[99]
*Mesorhizobium* sp., *Burkholderia* sp., *Pseudomonas* sp., *Rhizophagus irregularis*, *Funneliformis geosporum*, *Claroideoglomus claroideum*	*Cicer arietinum* L.	Drought	Increase of grain yield; and enhancement of crude protein content	[98]
*Klebsiella variicola*, *Glomus multisubtensum*, *Rhizophagus intraradices*	*Helianthus tuberosus* L.	-	Enhancement of plant growth and increase of tuber inulin content	[112]

## Data Availability

No new data were created or analyzed in this study. Data sharing is not applicable to this article.

## References

[1] Stockdale E.A., Shepherd M.A., Fortune S., Cuttle S.P. (2002). Soil fertility in organic farming systems–fundamentally different?. Soil Use Manag..

[2] Zhang H., Wang R., Chen S., Qi G., He Z., Zhao X. (2017). Microbial taxa and functional genes shift in degraded soil with bacterial wilt. Sci. Rep..

[3] Maximillian J., Brusseau M.L., Glenn E.P., Matthias A.D. (2019). Pollution and Environmental Perturbations in the Global System. Environmental and Pollution Science.

[4] Szparaga A., Kuboń M., Kocira S., Czerwińska E., Pawłowska A., Hara P., Kobus Z., Kwaśniewski D. (2019). Towards sustainable agriculture-agronomic and economic effects of biostimulant use in common bean cultivation. Sustainability.

[5] Mannino G., Gentile C., Ertani A., Serio G., Bertea C.M. (2021). Anthocyanins: Biosynthesis, Distribution, Ecological Role, and Use of Biostimulants to Increase Their Content in Plant Foods—A Review. Agriculture.

[6] European Commission (2020). Farm to Fork Strategy: For a fair, healthy and environmentally-friendly food system. DG SANTE/Unit Food Inf. Compos. Food Waste.

[7] Le Mire G., Nguyen M.L., Fassotte B., Du Jardin P., Verheggen F., Delaplace P., Haissam Jijakli M. (2016). Implementing plant biostimulants and biocontrol strategies in the agroecological management of cultivated ecosystems. A review. Biotechnol. Agron. Soc. Environ..

[8] Hellequin E., Monard C., Chorin M., Le Bris N., Daburon V., Klarzynski O., Binet F. (2020). Responses of active soil microorganisms facing to a soil biostimulant input compared to plant legacy effects. Sci. Rep..

[9] Campobenedetto C., Mannino G., Beekwilder J., Contartese V., Karlova R., Bertea C.M. (2021). The application of a biostimulant based on tannins affects root architecture and improves tolerance to salinity in tomato plants. Sci. Rep..

[10] Campobenedetto C., Grange E., Mannino G., Van Arkel J., Beekwilder J., Karlova R., Garabello C., Contartese V., Bertea C.M. (2020). A Biostimulant Seed Treatment Improved Heat Stress Tolerance during Cucumber Seed Germination by Acting on the Antioxidant System and Glyoxylate Cycle. Front. Plant Sci..

[11] European Commission (2019). The European Parliament and the Council of the European Union Regulation (EU) 2019/1009 of the European Parliament and of the Council of 5 June 2019 laying down rules on the making available on the market of EU fertilising products and amending Regulation (EC) No. 1069/2009 and (EC) No. 1107/2009 and repealing Regulat. Off. J. Eur. Union.

[12] Jacoby R., Peukert M., Succurro A., Koprivova A., Kopriva S. (2017). The role of soil microorganisms in plant mineral nutrition—Current knowledge and future directions. Front. Plant Sci..

[13] Romano I., Ventorino V., Pepe O. (2020). Effectiveness of Plant Beneficial Microbes: Overview of the Methodological Approaches for the Assessment of Root Colonization and Persistence. Front. Plant Sci..

[14] Copetta A., Lingua G., Berta G. (2006). Effects of three AM fungi on growth, distribution of glandular hairs, and essential oil production in Ocimum basilicum L. var. Genovese. Mycorrhiza.

[15] Chitarra W., Pagliarani C., Maserti B., Lumini E., Siciliano I., Cascone P., Schubert A., Gambino G., Balestrini R., Guerrieri E. (2016). Insights on the impact of arbuscular mycorrhizal symbiosis on tomato tolerance to water stress. Plant Physiol..

[16] Lally R.D., Galbally P., Moreira A.S., Spink J., Ryan D., Germaine K.J., Dowling D.N. (2017). Application of endophytic pseudomonas fluorescens and a bacterial consortium to brassica napus can increase plant height and biomass under greenhouse and field conditions. Front. Plant Sci..

[17] Zhang T., Hu Y., Zhang K., Tian C., Guo J. (2018). Arbuscular mycorrhizal fungi improve plant growth of Ricinus communis by altering photosynthetic properties and increasing pigments under drought and salt stress. Ind. Crops Prod..

[18] Boyer L.R., Brain P., Xu X.M., Jeffries P. (2015). Inoculation of drought-stressed strawberry with a mixed inoculum of two arbuscular mycorrhizal fungi: Effects on population dynamics of fungal species in roots and consequential plant tolerance to water deficiency. Mycorrhiza.

[19] Santander C., Sanhueza M., Olave J., Borie F., Valentine A., Cornejo P. (2019). Arbuscular Mycorrhizal Colonization Promotes the Tolerance to Salt Stress in Lettuce Plants through an Efficient Modification of Ionic Balance. J. Soil Sci. Plant Nutr..

[20] Remy W., Taylort T.N., Hass H., Kerp H. (1994). Four hundred-million-year-old vesicular arbuscular mycorrhizae. Proc. Natl. Acad. Sci. USA.

[21] Tedersoo L., Ko U., Bahram M., Sa S., Do M., May T., Ryberg M., Abarenkov K. (2018). High-level classification of the Fungi and a tool for evolutionary ecological analyses. Fungal Divers..

[22] Oehl F., Sieverding E., Palenzuela J., Ineichen K., da Silva G.A. (2011). Advances in Glomeromycota taxonomy and classification. IMA Fungus.

[23] Spatafora J.W., Chang Y., Benny G.L., Lazarus K., Smith E., Berbee M.L., Bonito G., Corradi N., Grigoriev I., Gryganskyi A. (2016). A phylum-level phylogenetic classification of zygomycete fungi based on genome-scale data. Mycologia.

[24] Giovannini L., Palla M., Agnolucci M., Avio L., Sbrana C., Turrini A., Giovannetti M. (2020). Arbuscular Mycorrhizal Fungi and Associated Microbiota as Plant Biostimulants: Research Strategies for the Selection of the Best Performing Inocula. Agronomy.

[25] Smith S.E., Read D. (2008). The symbionts forming arbuscular mycorrhizas. Mycorrhizal Symbiosis.

[26] Bulgarelli D., Schlaeppi K., Spaepen S., Van Themaat E.V.L., Schulze-Lefert P. (2013). Structure and functions of the bacterial microbiota of plants. Annu. Rev. Plant Biol..

[27] Pathania P., Rajta A., Singh P.C., Bhatia R. (2020). Role of plant growth-promoting bacteria in sustainable agriculture. Biocatal. Agric. Biotechnol..

[28] Mannino G., Nerva L., Gritli T., Novero M., Fiorilli V., Bacem M., Bertea C.M., Lumini E., Chitarra W., Balestrini R. (2020). Effects of Different Microbial Inocula on Tomato Tolerance to Water Deficit. Agronomy.

[29] Hashem A., Alqarawi A.A., Radhakrishnan R., Al-Arjani A.B.F., Aldehaish H.A., Egamberdieva D., Abd Allah E.F. (2018). Arbuscular mycorrhizal fungi regulate the oxidative system, hormones and ionic equilibrium to trigger salt stress tolerance in *Cucumis sativus* L.. Saudi J. Biol. Sci..

[30] Duc N.H., Csintalan Z., Posta K. (2018). Arbuscular mycorrhizal fungi mitigate negative effects of combined drought and heat stress on tomato plants. Plant Physiol. Biochem..

[31] Yasmin H., Nosheen A., Naz R., Bano A., Keyani R., Zea L. (2017). L-tryptophan-assisted PGPR-mediated induction of drought tolerance in maize (*Zea mays* L.). J. Plant Interact..

[32] Bárzana G., Aroca R., Ruiz-Lozano J.M. (2015). Localized and non-localized effects of arbuscular mycorrhizal symbiosis on accumulation of osmolytes and aquaporins and on antioxidant systems in maize plants subjected to total or partial root drying. Plant Cell Environ..

[33] Quiroga G., Erice G., Aroca R., Zamarreño Á.M., García-Mina J.M., Ruiz-Lozano J.M. (2018). Arbuscular mycorrhizal symbiosis and salicylic acid regulate aquaporins and root hydraulic properties in maize plants subjected to drought. Agric. Water Manag..

[34] Santos M.S., Nogueira M.A., Hungria M. (2021). Outstanding impact of azospirillum brasilense strains ab-v5 and ab-v6 on the brazilian agriculture: Lessons that farmers are receptive to adopt new microbial inoculants. Rev. Bras. Cienc. Solo.

[35] Asghari B., Khademian R., Sedaghati B. (2020). Plant growth promoting rhizobacteria (PGPR) confer drought resistance and stimulate biosynthesis of secondary metabolites in pennyroyal (*Mentha pulegium* L.) under water shortage condition. Sci. Hortic..

[36] Bhalerao R.P., Eklöf J., Ljung K., Marchant A., Bennett M., Sandberg G. (2002). Shoot-derived auxin is essential for early lateral root emergence in Arabidopsis seedlings. Plant J..

[37] Zou Y.N., Wang P., Liu C.Y., Ni Q.D., Zhang D.J., Wu Q.S. (2017). Mycorrhizal trifoliate orange has greater root adaptation of morphology and phytohormones in response to drought stress. Sci. Rep..

[38] Liu C.Y., Zhang F., Zhang D.J., Srivastava A., Wu Q.S., Zou Y.N. (2018). Mycorrhiza stimulates root-hair growth and IAA synthesis and transport in trifoliate orange under drought stress. Sci. Rep..

[39] Abd-Allah E.F., Hashem A., Alqarawi A.A., Bahkali A.H., Alwhibi M.S. (2015). Enhancing growth performance and systemic acquired resistance of medicinal plant *Sesbania sesban* (L.) Merr using arbuscular mycorrhizal fungi under salt stress. Saudi J. Biol. Sci..

[40] Rudawska M.L., Kieliszewska-Rokicka B. (1997). Mycorrhizal formation by Paxillus involutus strains in relation to their IAA-synthesizing activity. New Phytol..

[41] Chandra D., Srivastava R., Glick B.R., Sharma A.K. (2018). Drought-Tolerant Pseudomonas spp. Improve the Growth Performance of Finger Millet (*Eleusine coracana* (L.) Gaertn) under Non-Stressed and Drought-Stressed Conditions. Pedosphere.

[42] Huang X.F., Zhou D., Guo J., Manter D.K., Reardon K.F., Vivanco J.M. (2015). Bacillus spp.: From rainforest soil promote plant growth under limited nitrogen conditions. J. Appl. Microbiol..

[43] Glick B.R. (2014). Bacteria with ACC deaminase can promote plant growth and help to feed the world. Microbiol. Res..

[44] Radhakrishnan R., Hashem A., Abd Allah E.F. (2017). Bacillus: A biological tool for crop improvement through bio-molecular changes in adverse environments. Front. Physiol..

[45] Kang S.M., Khan A.L., Waqas M., You Y.H., Kim J.H., Kim J.G., Hamayun M., Lee I.J. (2014). Plant growth-promoting rhizobacteria reduce adverse effects of salinity and osmotic stress by regulating phytohormones and antioxidants in Cucumis sativus. J. Plant Interact..

[46] Okada K., Abe H., Arimura G.I. (2015). Jasmonates induce both defense responses and communication in monocotyledonous and dicotyledonous plants. Plant Cell Physiol..

[47] Bernardo L., Morcia C., Carletti P., Ghizzoni R., Badeck F.W., Rizza F., Lucini L., Terzi V. (2017). Proteomic insight into the mitigation of wheat root drought stress by arbuscular mycorrhizae. J. Proteom..

[48] Pedranzani H., Rodríguez-Rivera M., Gutiérrez M., Porcel R., Hause B., Ruiz-Lozano J.M. (2016). Arbuscular mycorrhizal symbiosis regulates physiology and performance of Digitaria eriantha plants subjected to abiotic stresses by modulating antioxidant and jasmonate levels. Mycorrhiza.

[49] De Vleesschauwer D., Xu J., Höfte M. (2014). Making sense of hormone-mediated defense networking: From rice to Arabidopsis. Front. Plant Sci..

[50] Turkan I. (2017). Emerging roles for ROS and RNS—Versatile molecules in plants. J. Exp. Bot..

[51] Kapoor D., Singh S., Kumar V., Romero R., Prasad R., Singh J. (2019). Antioxidant enzymes regulation in plants in reference to reactive oxygen species (ROS) and reactive nitrogen species (RNS). Plant Gene.

[52] Chiappero J., del Rosario Cappellari L., Alderete L.G.S., Palermo T.B., Banchio E. (2019). Plant growth promoting rhizobacteria improve the antioxidant status in Mentha piperita grown under drought stress leading to an enhancement of plant growth and total phenolic content. Ind. Crops Prod..

[53] Pauly N., Pucciariello C., Mandon K., Innocenti G., Jamet A., Baudouin E., Frendo P., Puppo A. (2006). Reactive oxygen and nitrogen species and glutathione: Key players in the legume-Rhizobium symbiosis. J. Exp. Bot..

[54] Ait-El-Mokhtar M., Laouane R.B., Anli M., Boutasknit A. (2019). Scientia Horticulturae Use of mycorrhizal fungi in improving tolerance of the date palm (*Phoenix dactylifera* L.) seedlings to salt stress. Sci. Hortic..

[55] Tian L., Shi S., Ma L., Zhou X., Luo S., Zhang J., Lu B., Tian C. (2019). The effect of Glomus intraradices on the physiological properties of Panax ginseng and on rhizospheric microbial diversity. J. Ginseng Res..

[56] Yasmeen T., Ahmad A., Arif M.S., Mubin M., Rehman K., Shahzad S.M., Iqbal S., Rizwan M., Ali S., Alyemeni M.N. (2020). Biofilm forming rhizobacteria enhance growth and salt tolerance in sunflower plants by stimulating antioxidant enzymes activity. Plant Physiol. Biochem..

[57] Nawaz A., Shahbaz M., Asadullah M., Imran A., Marghoob M.U., Imtiaz M., Mubeen F. (2020). Potential of Salt Tolerant PGPR in Growth and Yield Augmentation of Wheat (*Triticum aestivum* L.) under Saline Conditions. Front. Microbiol..

[58] Moreno-Galván A.E., Cortés-Patiño S., Romero-Perdomo F., Uribe-Vélez D., Bashan Y., Bonilla R.R. (2020). Proline accumulation and glutathione reductase activity induced by drought-tolerant rhizobacteria as potential mechanisms to alleviate drought stress in Guinea grass. Appl. Soil Ecol..

[59] Eke P., Kumar A., Sahu K.P., Wakam L.N., Sheoran N., Ashajyothi M., Patel A., Fekam F.B. (2019). Endophytic bacteria of desert cactus (*Euphorbia trigonas* Mill) confer drought tolerance and induce growth promotion in tomato (*Solanum lycopersicum* L.). Microbiol. Res..

[60] Zheng F.L., Liang S.M., Chu X.N., Yang Y.L., Wu Q.S. (2020). Mycorrhizal fungi enhance flooding tolerance of peach through inducing proline accumulation and improving root architecture. Plant Soil Environ..

[61] Gou W., Tian L., Ruan Z., Zheng P., Chen F., Zhang L., Cui Z., Zheng P., Li Z., Gao M. (2015). Accumulation of choline and glycinebetaine and drought stress tolerance induced in maize (*Zea mays*) by three plant growth promoting rhizobacteria (PGPR) strains. Pak. J. Bot..

[62] Tigka T., Ipsilantis I. (2020). Effects of sand dune, desert and field arbuscular mycorrhizae on lettuce (*Lactuca sativa*, L.) growth in a natural saline soil. Sci. Hortic..

[63] Li T., Lin G., Zhang X., Chen Y. (2014). Relative importance of an arbuscular mycorrhizal fungus (*Rhizophagus intraradices*) and root hairs in plant drought tolerance. Mycorrhiza.

[64] Zhang F., Zou Y., Wu Q. (2018). Scientia Horticulturae Quantitative estimation of water uptake by mycorrhizal extraradical hyphae in citrus under drought stress. Sci. Hortic..

[65] Ansari F.A., Ahmad I., Pichtel J. (2019). Growth stimulation and alleviation of salinity stress to wheat by the biofilm forming *Bacillus pumilus* strain FAB10. Appl. Soil Ecol..

[66] Bárzana G., Aroca R., Paz J.A., Chaumont F., Martinez-Ballesta M.C., Carvajal M., Ruiz-Lozano J.M. (2012). Arbuscular mycorrhizal symbiosis increases relative apoplastic water flow in roots of the host plant under both well-watered and drought stress conditions. Ann. Bot..

[67] Bárzana G., Aroca R., Bienert G.P., Chaumont F., Ruiz-Lozano J.M. (2014). New insights into the regulation of aquaporins by the arbuscular mycorrhizal symbiosis in maize plants under drought stress and possible implications for plant performance. Mol. Plant Microbe Interact..

[68] Yooyongwech S., Samphumphuang T., Tisarum R., Theerawitaya C., Cha-Um S. (2016). Arbuscular mycorrhizal fungi (AMF) improved water deficit tolerance in two different sweet potato genotypes involves osmotic adjustments via soluble sugar and free proline. Sci. Hortic..

[69] Navarro J.M., Pérez-Tornero O., Morte A. (2014). Alleviation of salt stress in citrus seedlings inoculated with arbuscular mycorrhizal fungi depends on the rootstock salt tolerance. J. Plant Physiol..

[70] Hazzoumi Z., Moustakime Y., Elharchli E., Joutei K.A. (2015). Effect of arbuscular mycorrhizal fungi (AMF) and water stress on growth, phenolic compounds, glandular hairs, and yield of essential oil in basil (*Ocimum gratissimum* L.). Chem. Biol. Technol. Agric..

[71] Colla G., Rouphael Y., Di Mattia E., El-Nakhel C., Cardarelli M. (2015). Co-inoculation of Glomus intraradices and Trichoderma atroviride acts as a biostimulant to promote growth, yield and nutrient uptake of vegetable crops. J. Sci. Food Agric..

[72] Kalaji H.M., Schansker G., Ladle R.J., Goltsev V. (2014). Frequently asked questions about in vivo chlorophyll fluorescence: Practical issues. Photosynth. Res..

[73] Bargaz A., Lyamlouli K., Chtouki M., Zeroual Y., Dhiba D. (2018). Soil Microbial Resources for Improving Fertilizers Efficiency in an Integrated Plant Nutrient Management System Soil Microbial Resources for Improving Fertilizers Efficiency in an Integrated plant nutrient management system. Front. Microbiol..

[74] Kumar A., Dames J.F., Gupta A., Sharma S., Gilbert J.A., Ahmad P. (2015). Current developments in arbuscular mycorrhizal fungi research and its role in salinity stress alleviation: A biotechnological perspective. Crit. Rev. Biotechnol..

[75] Bahadur A., Batool A., Nasir F., Jiang S., Mingsen Q., Zhang Q., Pan J., Liu Y., Feng H. (2019). Mechanistic insights into arbuscular mycorrhizal fungi-mediated drought stress tolerance in plants. Int. J. Mol. Sci..

[76] Garcia K., Zimmermann S.D. (2014). The role of mycorrhizal associations in plant potassium nutrition. Front. Plant Sci..

[77] Estrada B., Aroca R., Maathuis F.J.M., Barea J.M., Ruiz-Lozano J.M. (2013). Arbuscular mycorrhizal fungi native from a Mediterranean saline area enhance maize tolerance to salinity through improved ion homeostasis. Plant Cell Environ..

[78] Kour D., Rana K.L., Yadav A.N., Yadav N., Kumar M., Kumar V., Vyas P., Dhaliwal H.S., Saxena A.K. (2020). Microbial biofertilizers: Bioresources and eco-friendly technologies for agricultural and environmental sustainability. Biocatal. Agric. Biotechnol..

[79] Santos-Torres M., Romero-Perdomo F., Mendoza-Labrador J., Gutiérrez A.Y., Vargas C., Castro-Rincon E., Caro-Quintero A., Uribe-Velez D., Estrada-Bonilla G.A. (2021). Genomic and phenotypic analysis of rock phosphate-solubilizing rhizobacteria. Rhizosphere.

[80] Hii Y.S., Yen San C., Lau S.W., Danquah M.K. (2020). Isolation and characterisation of phosphate solubilizing microorganisms from peat. Biocatal. Agric. Biotechnol..

[81] Rijavec T., Lapanje A. (2016). Hydrogen cyanide in the rhizosphere: Not suppressing plant pathogens, but rather regulating availability of phosphate. Front. Microbiol..

[82] Begum N., Ahanger M.A., Zhang L. (2020). AMF inoculation and phosphorus supplementation alleviates drought induced growth and photosynthetic decline in Nicotiana tabacum by up-regulating antioxidant metabolism and osmolyte accumulation. Environ. Exp. Bot..

[83] Radzki W., Gutierrez Mañero F.J., Algar E., Lucas García J.A., García-Villaraco A., Ramos Solano B. (2013). Bacterial siderophores efficiently provide iron to iron-starved tomato plants in hydroponics culture. Antonie Leeuwenhoek.

[84] Georgieva T., Evstatieva Y., Savov V., Bratkova S., Nikolova D. (2018). Assessment of plant growth promoting activities of five rhizospheric Pseudomonas strains. Biocatal. Agric. Biotechnol..

[85] Shirinbayan S., Khosravi H., Jafar M. (2019). Alleviation of drought stress in maize (*Zea mays*) by inoculation with Azotobacter strains isolated from semi-arid regions. Appl. Soil Ecol..

[86] Ganugi P., Martinelli E., Lucini L. (2021). Microbial biostimulants as a sustainable approach to improve the functional quality in plant-based foods: A review. Curr. Opin. Food Sci..

[87] Sangiorgio D., Cellini A., Donati I., Pastore C., Onofrietti C., Spinelli F. (2020). Facing Climate Change: Application of Microbial Biostimulants to Mitigate Stress in Horticultural Crops. Agronomy.

[88] Hamid B., Zaman M., Farooq S., Fatima S., Sayyed R.Z., Baba Z.A., Sheikh T.A., Reddy M.S., El Enshasy H., Gafur A. (2021). Bacterial Plant Biostimulants: A Sustainable Way towards Improving Growth, Productivity, and Health of Crops. Sustainability.

[89] Ortiz N., Armada E., Duque E., Roldán A., Azcón R. (2015). Contribution of arbuscular mycorrhizal fungi and/or bacteria to enhancing plant drought tolerance under natural soil conditions: Effectiveness of autochthonous or allochthonous strains. J. Plant Physiol..

[90] Chenchouni H., Mekahlia M.N., Beddiar A. (2020). Effect of inoculation with native and commercial arbuscular mycorrhizal fungi on growth and mycorrhizal colonization of olive (*Olea europaea* L.). Sci. Hortic..

[91] Caravaca F., Barea J.M., Palenzuela J., Figueroa D., Alguacil M.M., Roldán A. (2003). Establishment of shrub species in a degraded semiarid site after inoculation with native or allochthonous arbuscular mycorrhizal fungi. Appl. Soil Ecol..

[92] Diagne N., Baudoin E., Svistoonoff S., Ouattara C., Diouf D., Kane A., Ndiaye C., Noba K., Bogusz D., Franche C. (2018). Effect of native and allochthonous arbuscular mycorrhizal fungi on Casuarina equisetifolia growth and its root bacterial community. Arid Land Res. Manag..

[93] Alguacil M.M., Caravaca F., Roldán A. (2005). Changes in rhizosphere microbial activity mediated by native or allochthonous AM fungi in the reafforestation of a Mediterranean degraded environment. Biol. Fertil. Soils.

[94] Knopf E., Blaschke H., Munch J.C. (2013). Improving moringa growth by using autochthonous and allochthonous arbuscular mycorrhizal fungi in Lake Victoria Basin. West Afr. J. Appl. Ecol..

[95] Benabdellah K., Abbas Y., Abourouh M., Aroca R., Azcón R. (2011). Influence of two bacterial isolates from degraded and non-degraded soils and arbuscular mycorrhizae fungi isolated from semi-arid zone on the growth of Trifolium repens under drought conditions: Mechanisms related to bacterial effectiveness. Eur. J. Soil Biol..

[96] Vyas P., Gulati A. (2009). Organic acid production in vitro and plant growth promotion in maize under controlled environment by phosphate-solubilizing fluorescent Pseudomonas. BMC Microbiol..

[97] Pereira S.I.A., Abreu D., Moreira H., Vega A., Castro P.M.L. (2020). Plant growth-promoting rhizobacteria (PGPR) improve the growth and nutrient use efficiency in maize (*Zea mays* L.) under water deficit conditions. Heliyon.

[98] Laranjeira S., Fernandes-Silva A., Reis S., Torcato C., Raimundo F., Ferreira L., Carnide V., Marques G. (2021). Inoculation of plant growth promoting bacteria and arbuscular mycorrhizal fungi improve chickpea performance under water deficit conditions. Appl. Soil Ecol..

[99] Diagne N., Ndour M., Djighaly P.I., Ngom D., Ngom M.C.N., Ndong G., Svistoonoff S., Cherif-Silini H. (2020). Effect of Plant Growth Promoting Rhizobacteria (PGPR) and Arbuscular Mycorrhizal Fungi (AMF) on Salt Stress Tolerance of Casuarina obesa (Miq.). Front. Sustain. Food Syst..

[100] Turnbull A.L., Campbell I., Lazarovits G. (2014). Resistance of bacterial communities in the potato rhizosphere to disturbance and its application to agroecology. Soil Biol. Biochem..

[101] Andrea D., Filippo-Herrera D., Muñoz-Ochoa M., Mireya Hernández-Herrera R., Hernández-Carmona G. (2019). Biostimulant activity of individual and blended seaweed extracts on the germination and growth of the mung bean. J. Appl. Phycol..

[102] Rouphael Y., Lucini L., Miras-Moreno B., Colla G., Bonini P., Cardarelli M. (2020). Metabolomic Responses of Maize Shoots and Roots Elicited by Combinatorial Seed Treatments with Microbial and Non-microbial Biostimulants. Front. Microbiol..

[103] Rouphael Y., Colla G. (2018). Synergistic biostimulatory action: Designing the next generation of plant biostimulants for sustainable agriculture. Front. Plant Sci..

[104] Agnolucci M., Avio L., Pepe A., Turrini A., Cristani C., Bonini P., Cirino V., Colosimo F., Ruzzi M., Giovannetti M. (2019). Bacteria associated with a commercial mycorrhizal inoculum: Community composition and multifunctional activity as assessed by illumina sequencing and culture-dependent tools. Front. Plant Sci..

[105] Nadeem S.M., Ahmad M., Zahir Z.A., Javaid A., Ashraf M. (2014). The role of mycorrhizae and plant growth promoting rhizobacteria (PGPR) in improving crop productivity under stressful environments. Biotechnol. Adv..

[106] Aalipour H., Nikbakht A., Etemadi N., Rejali F., Soleimani M. (2020). Biochemical response and interactions between arbuscular mycorrhizal fungi and plant growth promoting rhizobacteria during establishment and stimulating growth of Arizona cypress (*Cupressus arizonica* G.) under drought stress. Sci. Hortic..

[107] Visen A., Bohra M., Singh P.N., Srivastava P.C., Kumar S., Sharma A.K., Chakraborty B. (2017). Two pseudomonad strains facilitate AMF mycorrhization of litchi (*Litchi chinensis* Sonn.) and improving phosphorus uptake. Rhizosphere.

[108] Massa N., Cesaro P., Todeschini V., Capraro J., Scarafoni A., Cantamessa S., Copetta A., Anastasia F., Gamalero E., Lingua G. (2020). Selected autochthonous rhizobia, applied in combination with AM fungi, improve seed quality of common bean cultivated in reduced fertilization condition. Appl. Soil Ecol..

[109] Jeffries P., Gianinazzi S., Perotto S., Turnau K., Barea J.M. (2003). The contribution of arbuscular mycorrhizal fungi in sustainable maintenance of plant health and soil fertility. Biol. Fertil. Soils.

[110] Dal Cortivo C., Ferrari M., Visioli G., Lauro M., Fornasier F., Barion G., Panozzo A., Vamerali T. (2020). Effects of Seed-Applied Biofertilizers on Rhizosphere Biodiversity and Growth of Common Wheat (*Triticum aestivum* L.) in the Field. Front. Plant Sci..

[111] Barnawal D., Bharti N., Maji D., Chanotiya C.S., Kalra A. (2014). ACC deaminase-containing Arthrobacter protophormiae induces NaCl stress tolerance through reduced ACC oxidase activity and ethylene production resulting in improved nodulation and mycorrhization in Pisum sativum. J. Plant Physiol..

[112] Nacoon S., Jogloy S., Riddech N., Mongkolthanaruk W., Kuyper T.W., Boonlue S. (2020). Interaction between Phosphate Solubilizing Bacteria and Arbuscular Mycorrhizal Fungi on Growth Promotion and Tuber Inulin Content of *Helianthus tuberosus* L.. Sci. Rep..

[113] Dhawi F., Datta R., Ramakrishna W. (2017). Proteomics provides insights into biological pathways altered by plant growth promoting bacteria and arbuscular mycorrhiza in sorghum grown in marginal soil. Biochim. Biophys. Acta Proteins Proteom..

[114] Behrooz A., Vahdati K., Rejali F., Lotfi M., Sarikhani S., Leslie C. (2019). Arbuscular mycorrhiza and plant growth-promoting bacteria alleviate drought stress in walnut. HortScience.

[115] Moreira H., Pereira S.I.A., Vega A., Castro P.M.L., Marques A.P.G.C. (2020). Synergistic effects of arbuscular mycorrhizal fungi and plant growth-promoting bacteria benefit maize growth under increasing soil salinity. J. Environ. Manag..

[116] Cely M.V.T., Siviero M.A., Emiliano J., Spago F.R., Freitas V.F., Barazetti A.R., Goya E.T., Lamberti G.D.S., Dos Santos I.M.O., De Oliveira A.G. (2016). Inoculation of Schizolobium parahyba with mycorrhizal fungi and plant growth-promoting rhizobacteria increases wood yield under field conditions. Front. Plant Sci..

[117] Yakhin O.I., Lubyanov A.A., Yakhin I.A., Brown P.H. (2017). Biostimulants in plant science: A global perspective. Front. Plant Sci..

[118] Xu L., Geelen D. (2018). Developing biostimulants from agro-food and industrial by-products. Front. Plant Sci..

[119] Bharti N., Barnawal D., Wasnik K., Tewari S.K., Kalra A. (2016). Co-inoculation of Dietzia natronolimnaea and Glomus intraradices with vermicompost positively influences Ocimum basilicum growth and resident microbial community structure in salt affected low fertility soils. Appl. Soil Ecol..

[120] Ait-El-Mokhtar M., Baslam M., Ben-Laouane R., Anli M., Boutasknit A., Mitsui T., Wahbi S., Meddich A. (2020). Alleviation of Detrimental Effects of Salt Stress on Date Palm (*Phoenix dactylifera* L.) by the Application of Arbuscular Mycorrhizal Fungi and/or Compost. Front. Sustain. Food Syst..

[121] Susic M. (2016). Replenishing Humic Acids in Agricultural Soils. Agronomy.

[122] da Silva S.F., Olivares F.L., Canellas L.P. (2017). The biostimulant manufactured using diazotrophic endophytic bacteria and humates is effective to increase sugarcane yield. Chem. Biol. Technol. Agric..

[123] Torun H., Toprak B. (2020). Arbuscular Mycorrhizal Fungi and K-Humate Combined as Biostimulants: Changes in Antioxidant Defense System and Radical Scavenging Capacity in Elaeagnus angustifolia. J. Soil Sci. Plant Nutr..

[124] Ekin Z. (2019). Integrated Use of Humic Acid and Plant Growth Promoting Rhizobacteria to Ensure Higher Potato Productivity in Sustainable Agriculture. Sustainability.

[125] Ertani A., Francioso O., Tinti A., Schiavon M., Pizzeghello D., Nardi S. (2018). Evaluation of seaweed extracts from Laminaria and *Ascophyllum nodosum* spp. as biostimulants in *Zea mays* L. Using a combination of chemical, biochemical and morphological approaches. Front. Plant Sci..

[126] Kapoore R.V., Wood E.E., Llewellyn C.A. (2021). Algae biostimulants: A critical look at microalgal biostimulants for sustainable agricultural practices. Biotechnol. Adv..

[127] González-González M.F., Ocampo-Alvarez H., Santacruz-Ruvalcaba F., Sánchez-Hernández C.V., Casarrubias-Castillo K., Becerril-Espinosa A., Castañeda-Nava J.J., Hernández-Herrera R.M. (2020). Physiological, Ecological, and Biochemical Implications in Tomato Plants of Two Plant Biostimulants: Arbuscular Mycorrhizal Fungi and Seaweed Extract. Front. Plant Sci..

[128] Kopta T., Pavlíková M., Sȩkara A., Pokluda R., Maršálek B. (2018). Effect of bacterial-algal biostimulant on the yield and internal quality of Lettuce (*Lactuca sativa* L.) produced for spring and summer crop. Not. Bot. Horti Agrobot. Cluj Napoca.

[129] Moradtalab N., Hajiboland R., Aliasgharzad N., Hartmann T.E., Neumann G. (2019). Silicon and the Association with an Arbuscular-Mycorrhizal Fungus (*Rhizophagus clarus*) Mitigate the Adverse Effects of Drought Stress on Strawberry. Agronomy.

[130] Pavlovic J., Samardzic J., Maksimovi V., Timotijevic G., Stevic N., Laursen K.H., Hansen T.H., Husted S., Schjoerring J.K., Liang Y. (2013). Silicon alleviates iron deficiency in cucumber by promoting mobilization of iron in the root apoplast. New Phytol..

[131] Vega I., Nikolic M., Pontigo S., Godoy K., Mora M.D.L.L., Cartes P. (2019). Silicon Improves the Production of High Antioxidant or Structural Phenolic Compounds in Barley Cultivars under Aluminum Stress. Agronomy.

[132] Kim Y.H., Khan A.L., Waqas M., Lee I.J. (2017). Silicon regulates antioxidant activities of crop plants under abiotic-induced oxidative stress: A review. Front. Plant Sci..

[133] Ertani A., Francioso O., Ferrari E., Schiavon M., Nardi S. (2018). Spectroscopic-Chemical Fingerprint and Biostimulant Activity of a Protein-Based Product in Solid Form. Molecules.

[134] Kumar V., Singh S.P., Raha P. (2018). Organic sources use of amino acids based biostimulants and irrigation schedule on yield: Water use efficiency relationship on potato tuber. J. Pharmacogn. Phytochem..

[135] Popko M., Michalak I., Wilk R., Gramza M., Chojnacka K., Górecki H. (2018). Effect of the New Plant Growth Biostimulants Based on Amino Acids on Yield and Grain Quality of Winter Wheat. Molecules.

[136] Tewari S., Pooniya V., Sharma S. (2020). Next generation bioformulation prepared by amalgamating Bradyrhizobium, cell free culture supernatant, and exopolysaccharides enhances the indigenous rhizospheric rhizobial population, nodulation, and productivity of pigeon pea. Appl. Soil Ecol..

[137] Ahmad F., Ahmad I., Khan M.S. (2008). Screening of free-living rhizospheric bacteria for their multiple plant growth promoting activities. Microbiol. Res..

[138] Bradáčová K., Sittinger M., Tietz K., Neuhäuser B., Kandeler E., Berger N., Ludewig U., Neumann G. (2019). Maize inoculation with microbial consortia: Contrasting effects on rhizosphere activities, nutrient acquisition and early growth in different soils. Microorganisms.

[139] Thomloudi E.E., Tsalgatidou P.C., Douka D., Spantidos T.N., Dimou M., Venieraki A., Katinakis P. (2019). Multistrain versus single-strain plant growth promoting microbial inoculants-The compatibility issue. Hell. Plant Prot. J..

[140] Raaijmakers J.M., De Bruijn I., Nybroe O., Ongena M. (2010). Natural functions of lipopeptides from Bacillus and Pseudomonas: More than surfactants and antibiotics. FEMS Microbiol. Rev..

[141] Couillerot O., Combes-Meynet E., Pothier F., Challita E., Bellvert F., Comte G., Moe Y. (2011). The role of the antimicrobial compound 2,4-diacetylphloroglucinol in the impact of biocontrol Pseudomonas fluorescens F113 on Azospirillum brasilense phytostimulators. Microbiology.

[142] Pangesti N., Vandenbrande S., Pineda A., Dicke M., Raaijmakers J.M., Van Loon J.J.A. (2017). Antagonism between two root-associated beneficial Pseudomonas strains does not affect plant growth promotion and induced resistance against a leaf-chewing herbivore. FEMS Microbiol. Ecol..

[143] Engelmoer D.J., Behm J.E., Kiers E.T. (2014). Intense competition between arbuscular mycorrhizal mutualists in an in vitro root microbiome negatively affects total fungal abundance. Mol. Ecol..

[144] Trabelsi D., Mhamdi R. (2013). Microbial Inoculants and Their Impact on Soil Microbial Communities: A Review. BioMed Res. Int..

[145] Mannino G., Campobenedetto C., Vigliante I., Contartese V., Gentile C., Bertea C.M. (2020). The Application of a Plant Biostimulant Based on Seaweed and Yeast Extract Improved Tomato Fruit Development and Quality. Biomolecules.

[146] Sharma N., Singhvi R. (2017). Effects of chemical fertilizers and pesticides on human health and environment: A review. Int. J. Agric. Environ. Biotechnol..

[147] Dickson-Spillmann M., Siegrist M., Keller C. (2011). Attitudes toward chemicals are associated with preference for natural food. Food Qual. Prefer..

[148] Campobenedetto C., Mannino G., Agliassa C., Acquadro A., Contartese V., Garabello C., Bertea C.M. (2020). Transcriptome Analyses and Antioxidant Activity Profiling Reveal the Role of a Lignin-Derived Biostimulant Seed Treatment in Enhancing Heat Stress Tolerance in Soybean. Plants.

[149] Rodrigues M., Baptistella J.L.C., Horz D.C., Bortolato L.M., Mazzafera P. (2020). Organic plant biostimulants and fruit quality—A review. Agronomy.

[150] Boca G.D. (2021). Factors influencing consumer behavior in sustainable fruit and vegetable consumption in maramures county, Romania. Sustainability.

[151] Huang W.-S., Kuo H.-Y., Tung S.-Y., Chen H.-S. (2021). Assessing Consumer Preferences for Suboptimal Food: Application of a Choice Experiment in Citrus Fruit Retail. Foods.

[152] Parada J., Valenzuela T., Gómez F., Tereucán G., García S., Cornejo P., Winterhalter P., Ruiz A. (2019). Effect of fertilization and arbuscular mycorrhizal fungal inoculation on antioxidant profiles and activities in Fragaria ananassa fruit. J. Sci. Food Agric..

[153] Morais M.C., Mucha Â., Ferreira H., Gonçalves B., Bacelar E., Marques G. (2019). Comparative study of plant growth-promoting bacteria on the physiology, growth and fruit quality of strawberry. J. Sci. Food Agric..

[154] Moreno-Salazar R., Sánchez-García I., Chan-Cupul W., Ruiz-Sánchez E., Hernández-Ortega H.A., Pineda-Lucatero J., Figueroa-Chávez D. (2020). Plant growth, foliar nutritional content and fruit yield of Capsicum chinense biofertilized with Purpureocillium lilacinum under greenhouse conditions. Sci. Hortic..

[155] Hart M., Ehret D.L., Krumbein A., Leung C., Murch S., Turi C., Franken P. (2015). Inoculation with arbuscular mycorrhizal fungi improves the nutritional value of tomatoes. Mycorrhiza.

[156] Golubkina N., Gomez L.D., Kekina H., Cozzolino E., Simister R., Tallarita A., Torino V., Koshevarov A., Cuciniello A., Maiello R. (2020). Joint Selenium-Iodine Supply and Arbuscular Mycorrhizal Fungi Inoculation Affect Yield and Quality of Chickpea Seeds and Residual Biomass. Plants.

[157] Todeschini V., AitLahmidi N., Mazzucco E., Marsano F., Gosetti F., Robotti E., Bona E., Massa N., Bonneau L., Marengo E. (2018). Impact of Beneficial Microorganisms on Strawberry Growth, Fruit Production, Nutritional Quality, and Volatilome. Front. Plant Sci..

[158] Inculet C.-S., Mihalache G., Sellitto V.M., Hlihor R.-M., Stoleru V. (2019). The effects of a microorganisms-based commercial product on the morphological, biochemical and yield of tomato plants under two different water regimes. Microorganisms.

[159] de Almeida T.G., Alves M.B.R., Batissaco L., Torres M.A., de Andrade A.F.C., Mingoti R.D., de Arruda R.P., Celeghini E.C.C. (2019). Does low-level laser therapy on degenerated ovine testes improve post-thawed sperm characteristics?. Lasers Med. Sci..

[160] Sabatino L., Iapichino G., Consentino B.B., D’Anna F., Rouphael Y. (2020). Rootstock and Arbuscular Mycorrhiza Combinatorial Effects on Eggplant Crop Performance and Fruit Quality under Greenhouse Conditions. Agronomy.

[161] Chabay I. (2018). Land Degradation and Restoration.

[162] Nachtergaele F.O., Petri M., Biancalani R., Lal R., Stewart B.A. (2012). Land degradation. World Soil Resources and Food Security.

[163] Debonne N., van Vliet J., Metternicht G., Verburg P. (2021). Agency shifts in agricultural land governance and their implications for land degradation neutrality. Glob. Environ. Chang..

[164] Bai Y.-C., Chang Y.-Y., Hussain M., Lu B., Zhang J.-P., Song X.-B., Lei X.-S., Pei D. (2020). Soil Chemical and Microbiological Properties Are Changed by Long-Term Chemical Fertilizers that Limit Ecosystem Functioning. Microorganisms.

[165] Yang G., Roy J., Veresoglou S.D., Rillig M.C. (2021). Soil biodiversity enhances the persistence of legumes under climate change. New Phytol..

[166] Lehman R., Cambardella C., Stott D., Acosta-Martinez V., Manter D., Buyer J., Maul J., Smith J., Collins H., Halvorson J. (2015). Understanding and Enhancing Soil Biological Health: The Solution for Reversing Soil Degradation. Sustainability.

[167] Luján Soto R., Martínez-Mena M., Cuéllar Padilla M., de Vente J. (2021). Restoring soil quality of woody agroecosystems in Mediterranean drylands through regenerative agriculture. Agric. Ecosyst. Environ..

[168] Wang C., Liu D., Bai E. (2018). Decreasing soil microbial diversity is associated with decreasing microbial biomass under nitrogen addition. Soil Biol. Biochem..

[169] Zhou Z., Wang C., Luo Y. (2020). Meta-analysis of the impacts of global change factors on soil microbial diversity and functionality. Nat. Commun..

[170] Castro H.F., Classen A.T., Austin E.E., Norby R.J., Schadt C.W. (2010). Soil microbial community responses to multiple experimental climate change drivers. Appl. Environ. Microbiol..

[171] Zhang Y., Dong S., Gao Q., Liu S., Zhou H., Ganjurjav H., Wang X. (2016). Climate change and human activities altered the diversity and composition of soil microbial community in alpine grasslands of the Qinghai-Tibetan Plateau. Sci. Total Environ..

[172] Qiu L., Zhang Q., Zhu H., Reich P.B., Banerjee S., van der Heijden M.G.A., Sadowsky M.J., Ishii S., Jia X., Shao M. (2021). Erosion reduces soil microbial diversity, network complexity and multifunctionality. ISME J..

[173] Trivedi C., Delgado-Baquerizo M., Hamonts K., Lai K., Reich P.B., Singh B.K. (2019). Losses in microbial functional diversity reduce the rate of key soil processes. Soil Biol. Biochem..

[174] Kallenbach C.M., Frey S.D., Grandy A.S. (2016). Direct evidence for microbial-derived soil organic matter formation and its ecophysiological controls. Nat. Commun..

[175] Zheng T., Miltner A., Liang C., Nowak K.M., Kästner M. (2021). Turnover of gram-negative bacterial biomass-derived carbon through the microbial food web of an agricultural soil. Soil Biol. Biochem..

[176] Liang C., Amelung W., Lehmann J., Kästner M. (2019). Quantitative assessment of microbial necromass contribution to soil organic matter. Glob. Chang. Biol..

[177] Buckeridge K.M., Mason K.E., McNamara N.P., Ostle N., Puissant J., Goodall T., Griffiths R.I., Stott A.W., Whitaker J. (2020). Environmental and microbial controls on microbial necromass recycling, an important precursor for soil carbon stabilization. Commun. Earth Environ..

[178] De Vries F.T., Griffiths R.I., Knight C.G., Nicolitch O., Williams A. (2020). Harnessing rhizosphere microbiomes for drought-resilient crop production. Science.

[179] Prudent M., Dequiedt S., Sorin C., Girodet S., Nowak V., Duc G., Salon C., Maron P.A. (2020). The diversity of soil microbial communities matters when legumes face drought. Plant Cell Environ..

[180] Baldi E., Gioacchini P., Montecchio D., Mocali S., Antonielli L., Masoero G., Toselli M., Sanjuan S. (2021). Effect of Biofertilizers Application on Soil Biodiversity and Litter Degradation in a Commercial Apricot Orchard. Agronomy.

